# Age-related deficits in dip-listening evident for isolated sentences but not for spoken stories

**DOI:** 10.1038/s41598-022-09805-6

**Published:** 2022-04-07

**Authors:** Vanessa C. Irsik, Ingrid S. Johnsrude, Björn Herrmann

**Affiliations:** 1 Department of Psychology & The Brain and Mind Institute, The University of Western Ontario, London, ON N6A 3K7 Canada; 2 School of Communication and Speech Disorders, The University of Western Ontario, London, ON N6A 5B7 Canada; 3 Rotman Research Institute, Baycrest, Toronto, ON M6A 2E1 Canada; 4 Department of Psychology, University of Toronto, Toronto, ON M5S 1A1 Canada

**Keywords:** Auditory system, Cognitive ageing, Human behaviour

## Abstract

Fluctuating background sounds facilitate speech intelligibility by providing speech ‘glimpses’ (masking release). Older adults benefit less from glimpses, but masking release is typically investigated using isolated sentences. Recent work indicates that using engaging, continuous speech materials (e.g., spoken stories) may qualitatively alter speech-in-noise listening. Moreover, neural sensitivity to different amplitude envelope profiles (ramped, damped) changes with age, but whether this affects speech listening is unknown. In three online experiments, we investigate how masking release in younger and older adults differs for masked sentences and stories, and how speech intelligibility varies with masker amplitude profile. Intelligibility was generally greater for damped than ramped maskers. Masking release was reduced in older relative to younger adults for disconnected sentences, and stories with a randomized sentence order. Critically, when listening to stories with an engaging and coherent narrative, older adults demonstrated equal or greater masking release compared to younger adults. Older adults thus appear to benefit from ‘glimpses’ as much as, or more than, younger adults when the speech they are listening to follows a coherent topical thread. Our results highlight the importance of cognitive and motivational factors for speech understanding, and suggest that previous work may have underestimated speech-listening abilities in older adults.

## Introduction

Speech sounds are characterized by low frequency amplitude fluctuations that are not only critical for speech intelligibility in quiet^1–3^, but are also a useful cue for separating speech from background masking sounds^4–6^. Aging is associated with a decline in processing temporal auditory features^7–10^, such as the fluctuating speech envelope, which may be part of the reason older adults frequently struggle to understand speech when background masking sounds are present^11,12^. We recently demonstrated that cortical sensitivity to a sound’s envelope fluctuations with different temporal profiles differs between younger and older adults^13^, raising the possibility that particular envelope profiles may alter how effectively a masker occludes target speech for older adults. Furthermore, we have also shown that engaging spoken narratives qualitatively alter the speech-listening experience when background noise is present^14^, compared to the disconnected sentence-length utterances that are typically used in speech research^15–20^. In the current study we investigate how the temporal profile of the background masker influences intelligibility of isolated sentences compared to narrative stories in younger and older adults.

For most younger healthy individuals, amplitude variations in background masking sounds facilitate speech intelligibility compared to an energetically matched masker with a flat envelope. Fluctuating maskers are thought to enable a listener to perceive ‘glimpses’ of the target speech (c.f., “listening in the dips”)^21^. The intelligibility benefit for fluctuating over energetically matched, flat-envelope maskers is a type of “release from masking”^4–6,22–26^. Whereas younger listeners derive a robust benefit from fluctuating maskers, older adults have been consistently demonstrated to either gain no benefit to intelligibility, or a reduced benefit (compared to younger people)^16,19,23,24,27–31^. These findings may partially result from reduced audibility which reduces the effective amplitude-modulation depth^20,24^, and therefore the opportunity for ‘glimpses’ of the target. However, controlling for audibility has not resulted in restoration of the release-from-masking effect in all older individuals^18,23,28,32^. An age-related decline in temporal resolution in the auditory system may also contribute, as reduced temporal resolution can result in increased susceptibility to forward masking^28,31^, thereby reducing the available ‘glimpses’ of the target (see also the contribution of temporal fine structure^21,26,30,33–35^).

The amplitude envelope of speech is temporally dynamic: it varies in the rate of rise (attack) and fall (decay) over time^36^. Sensitivity to the shape of amplitude envelopes is important for identifying and discriminating between different consonants (e.g., /pa/ versus /ta/)^3^. Previous research in younger and older human listeners^13^, and in rats^37^, indicates that aging is associated with a relative increase in neural sensitivity to sounds with damped (sharp attack and gradual decay) compared to ramped (gradual attack and sharp decay) envelope shapes. Moreover, enhanced neural sensitivity to amplitude modulations in sounds has been linked to reduced speech intelligibility when the background sound is amplitude modulated^38–40^. Enhanced neural sensitivity to amplitude envelopes may distort envelope cues^41,42^ or, when part of a masking stream of sound, distract an older listener and interfere with comprehension^38,43–45^. Here, we examine whether the temporal envelope profile of the masker affects susceptibility to masking in young and older listeners. Given that older listeners exhibit greater cortical sensitivity to damped sounds^13,37^, we anticipate that older listeners will obtain less masking release when target speech is masked by sound with a damped, compared to ramped, amplitude envelope.

Studies that investigate phenomena affecting speech understanding, such as release from masking, generally use brief, disconnected speech utterances, like isolated sentences^15–20^. Such utterances typically lack a narrative thread and may not be very interesting to the listener. In everyday listening situations, sentences are not typically disconnected. Instead, conversational speech frequently contains inter-related narrated elements, such as stories about past events^46–58^. Narrated descriptions of past events have been reported to occur as often as 5.4 times per hour^55^. While the structure of a spoken story or narrative can vary based on the conversational circumstances^50^, narrated speech generally follows a topical thread and is contextually rich. The presence of speech context and an overarching topical thread in spoken narratives may support speech understanding and ongoing attention as sentence-level context has been shown to facilitate word identification in noise for both younger and older listeners^17,59–63^.

In addition, cognitive control research suggests that motivation is key to the investment of cognitive resources^64–66^, such as when trying to understand speech masked by background sounds^67–71^ and an increasing body of work thus focuses on using enjoyable (i.e., motivating) stories or narratives to investigate speech listening^72–83^. For example, although moderate speech masking decreases speech intelligibility and increases listening effort in young normally hearing listeners, story absorption and enjoyment are only minimally affected^14^. Critically, when a listener is motivated to understand (e.g., when listening to engaging spoken stories), they may be engaging in a different way that may promote intelligibility, compared to when they are less motivated to understand. This may particularly be the case for older adults who may not engage in tasks with low personal relevance in order to conserve resources for more personally relevant tasks^84,85^. Engaging speech materials may thus reveal qualitative differences between age groups, particularly in the extent to which ‘speech glimpses’ or ‘dip listening’ facilitates intelligibility.

In three behavioral experiments, we use masked disconnected sentences and engaging spoken stories in order to examine how the type of speech utterance and masker temporal profile affects speech intelligibility in younger and older adults. We utilize 12-talker babble masking noise with different amplitude envelopes. The envelope could either be unmodulated (i.e., relatively flat), modulated with a damped temporal profile, or modulated with a ramped temporal profile. In Experiment 1, we examine the effect of different masker modulation types and age on intelligibility using isolated sentences. In Experiment 2, we conduct a similar investigation with an engaging story as the target speech. Given that the procedures and speech materials differ between Experiment 1 and 2, we conduct Experiment 3 using identical procedures and materials for both disconnected-sentence and story conditions.

## Results

### Experiment 1: release from masking is reduced in older adults for disconnected sentences

In Experiment 1, we investigate how the amplitude envelope type (modulated vs unmodulated) and envelope shape (damped vs. ramped) affect speech intelligibility using a sentence-based intelligibility paradigm. We use similar procedures to those previously used to study release from masking^19,20,24,27,28^ in order to (a) replicate previous observations that older adults benefit less from a modulated over an unmodulated masker compared to younger adults, and (b) examine whether the shape of the modulation (damped or ramped) influences the magnitude of release from masking observed.

The experiment was conducted online using Amazon Mechanical Turk (MTurk; https://www.mturk.com/) and Cloud Research (previously TurkPrime^86^) for recruitment and Pavlovia (https://pavlovia.org/) to host the experiment. Younger (mean: 35.9 years; age-range: 18–49 years; 39 males, 29 females, 1 non-binary) and older adults (mean: 59.6 years; age-range: 50–71 years; 31 males, 37 females) without reported hearing or neurological issues (self-report) participated in the experiment. Based on previous work^87^ and results from a separate project (see Supplemental Document), we estimate that older adults in the current study had about 7 dB HL higher audiometric pure-tone average thresholds compared to younger adults. The estimation further suggests that approximately 25% of our older adult sample may have a minor hearing impairment (see Supplemental Document), as would be expected from a group of older adults recruited from the community.

**Figure 1:**
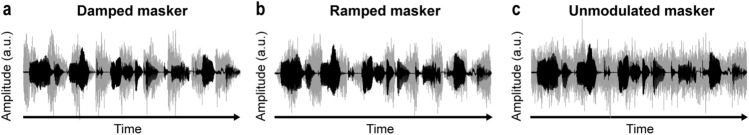
Stimulus design for Experiment 1. Participants listened to sentences (black) to which a 12-talker babble was added with varied SNR levels (grey). Note that for the purpose of clarity, we depict sentence and masker separately. Babble noise was amplitude modulated at a rate of 4-Hz with (**a**) a damped (**b**) a ramped envelope shape; or was (**c**) unmodulated.

**Figure 2:**
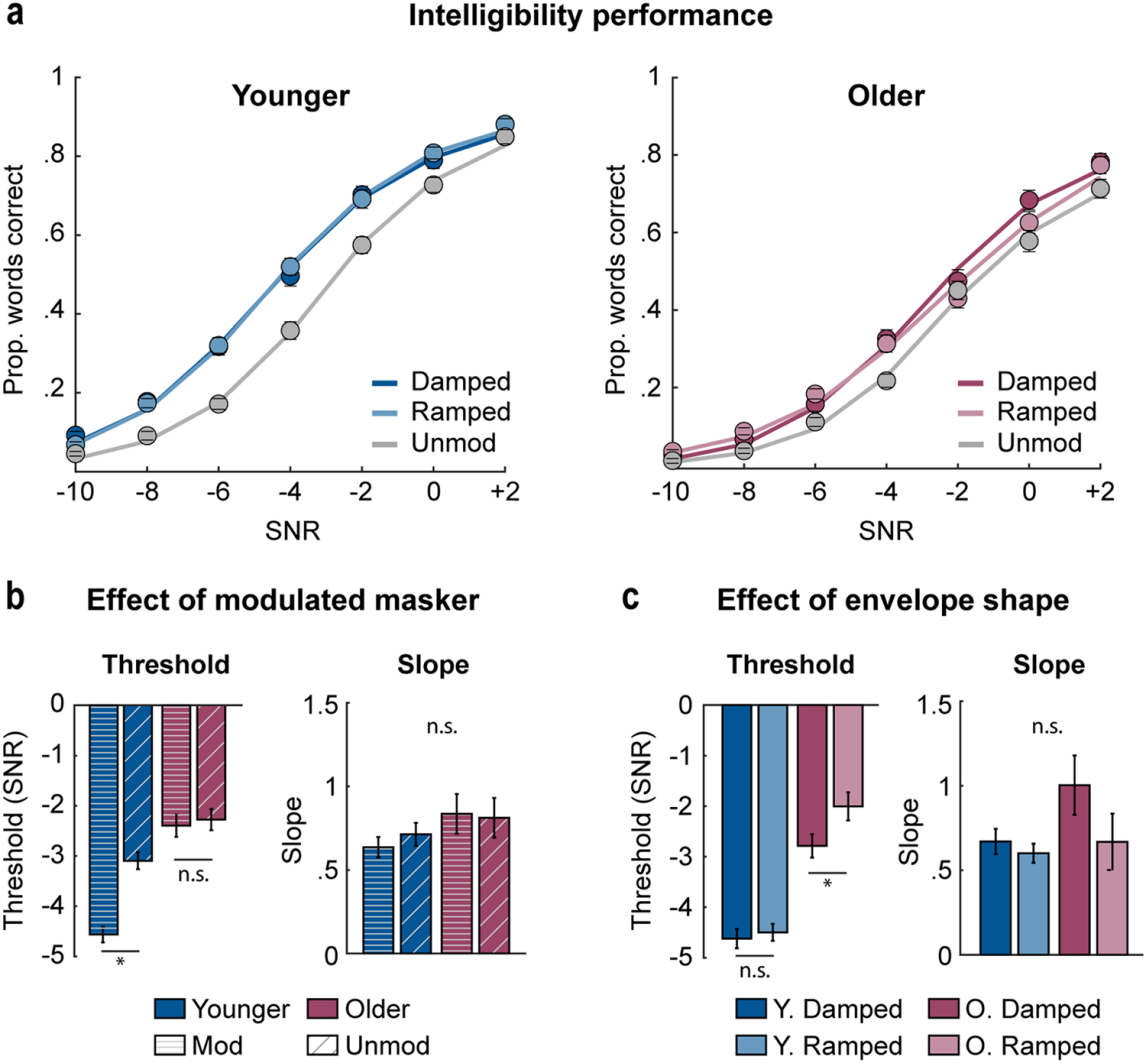
Intelligibility results for Experiment 1. (**a**) Mean proportion of correctly reported words plotted as a function of SNR (− 10, − 8, − 6, − 4, − 2, 0, + 2 dB) for younger (left) and older adults (right), and for envelope conditions (damped, ramped, unmodulated). Colored lines correspond to a logistic function fit to the proportion of correct words reported. (**b**) Mean threshold (left) and slope (right) coefficients from logistic function fits are plotted for the different modulation types (modulated [averaged across ramped and damped], unmodulated) and age groups (younger, older). (**c**) Mean threshold (left) and slope (right) coefficients are plotted for the different masker envelope shapes (damped, ramped) and age groups (younger, older). Error bars reflect the standard error of the mean. *p < 0.05.

During the task, participants listened to disconnected sentences and, after each sentence, typed the words they heard into a text box. A 12-talker babble masker was added to each sentence, and the signal-to-noise ratio (SNR levels: − 10, − 8,  −  6, − 4, − 2, 0, + 2 dB) and temporal profile of the amplitude envelope of the masker (unmodulated, 4-Hz amplitude-modulated: damped, ramped) were varied (Fig. 1). We calculated the proportion of correctly reported words for each envelope condition and SNR, fit a logistic function to the mean performance data (Fig. 2a), and analyzed the speech reception threshold (SNR associated with 50% correctly reported words) and slope.

To examine whether the magnitude of masking release differs between age groups, we compared the threshold and slope from the logistic function fits between modulated (unweighted average across ramped and damped shapes) and unmodulated masker conditions and between younger and older adults. Speech reception thresholds for sentences with a modulated masker were lower than for unmodulated maskers [effect of modulation type: *F*_1,135_ = 27.9, *p* = 5 × 10^–7^, η^2^p = 0.17], consistent with previous reports of release from masking^6,16,26–28^ (we replicated this effect in a group of younger adults in a laboratory setting; Supplemental Document). Thresholds were also lower for younger compared to older adults [effect of age group: *F*_1,135_ = 43.91, *p* = 8 × 10^–10^, η^2^p = 0.25], consistent with older adults having more difficulty understanding speech in noise^12,88^. We further observed a significant modulation type × age group interaction [*F*_1,135_ = 20.02, *p* = 2 × 10^–5^, η^2^p = 0.13; Fig. 2b left]: Speech intelligibility was better for modulated compared to unmodulated maskers in younger individuals [*t*_68_ = -8.78, *p*_*FDR*_ = 2 × 10^–12^, r_e_ = 0.73], whereas no difference was found for older adults [*p*_*FDR*_ = 0.63]. This is consistent with previous research^16,19,27,28,31^ indicating that older adults experience reduced release from masking—for fluctuating compared to flat masker envelopes—relative to younger adults. Older adults thus do not appear to utilize glimpse listening, at least for the disconnected sentences used here. No significant differences were observed when analyzing slopes [*F* < 2, *p* > 0.17, η^2^p < 0.01, Fig. 2b right].

For the analysis of different envelope shapes (damped vs. ramped), we observed lower thresholds for damped compared to ramped envelopes [effect of envelope shape: *F*_1,135_ = 8.65, *p* = 0.004, η^2^p = 0.06], and a significant envelope shape × age group interaction [*F*_1,135_ = 4.55, *p* = 0.035, η^2^p = 0.03; Fig. 2c left]. Speech intelligibility thresholds were better (lower) for damped compared to ramped envelope shapes for older adults [*t*_67_ = − 3.05, *p*_*FDR*_ = 0.007, r_e_ = 0.35], but not younger adults [*p*_*FDR*_ = 0.473]. No significant differences were observed for slope [*F* < 3, *p* > 0.11, η^2^p < 0.02; Fig. 2c right].

The results of Experiment 1 parallel previous findings on the effect of amplitude modulations on speech intelligibility^16,25,27,28,31,89^. We show that older individuals benefit less from a modulated over an unmodulated masker, compared to younger participants. We also observed that older, but not younger, listeners benefited when the babble background was modulated with a damped compared to a ramped envelope shape. This speech intelligibility benefit for damped temporal profiles is inconsistent with a recently proposed hypothesis based on electrophysiological work: Older adults demonstrate larger cortical responses to damped compared to ramped sounds^13^, and larger cortical responses to amplitude modulations have been linked to poorer speech intelligibility^38–40^. Hence, we anticipated that damped babble would interfere more, not less, with the target speech. Instead, increased cortical responsivity to the damped compared to ramped masker may strengthen predictability of modulation phase, facilitating speech ‘glimpsing’.

The short, disconnected sentences used in Experiment 1 are similar to those commonly used in speech intelligibility and masking release research. However, disconnected sentences without a topical thread may be less common in everyday listening situations, where speech is commonly continuous and contains narrated elements^46–58^. Experiment 2 was designed to investigate whether the effects obtained in Experiment 1 generalize to materials that resemble listening situations with more structured narrated elements, such as spoken stories about life events.

### Experiment 2: masking release is greater for older compared to younger adults during story listening

In Experiment 2, we investigate how the amplitude envelope type (modulated vs unmodulated) and envelope shape (damped vs. ramped) affect speech intelligibility while younger (mean: 30.1 years; age-range: 19–39 years; 37 males, 30 females) and older individuals (mean: 64.4 years; age-range: 53–80 years; 29 males, 41 females) without reported hearing or neurological issues listen to stories. Participant recruitment and testing was conducted using online platforms, as in Experiment 1. We selected a ~ 13-min spoken story from the story-telling podcast The Moth (https://themoth.org), where individuals tell stories about interesting life events. Stories are intended to be engaging and enjoyable, and are increasingly used in experimental research to study engagement with speech^14,90–93^.

**Figure 3:**
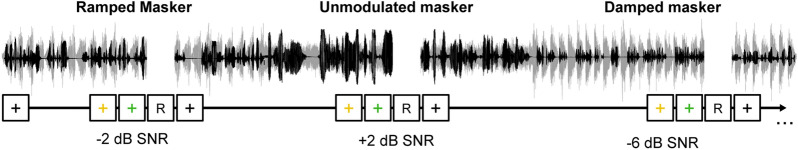
Stimulus design for Experiment 2. Participants listened to a spoken story (black) masked with 12-talker babble noise (grey). The amplitude envelope (damped, ramped, unmodulated) and SNR (− 6, − 2, + 2, Clear) pseudo-randomly varied every 16-s. A fixation cross was displayed on the computer screen throughout the story and changed colors to communicate which parts of the story participants would need to report. The fixation cross turned yellow 2-s prior to the beginning of a test phrase/sentence, cueing the participant to prepare for intelligibility testing, and turned green at the start of the test phrase/sentence to indicate which phrase/sentence they should report back. The story paused with the offset of the test phrase/sentence, at which point participants would report back the phrase/sentence. The story resumed once a response was submitted.

The story was masked by 12-talker babble with different amplitude envelopes (unmodulated, 4-Hz modulated: damped, ramped) and different signal-to-noise ratios (SNRs: − 6, − 2, + 2 dB, Clear). Masker type and SNR changed approximately every 16 s (Fig. 3). The story paused pseudorandomly (approximately every 5–20 s), and participants were asked to report the last phrase/sentence that was spoken by typing into a textbox. A visual cue directed participants exactly which words they should report back (Fig. 3). We calculated the proportion of correctly reported words for each envelope condition (damped, ramped, unmodulated) and SNR (− 6, − 2, + 2 dB, Clear) and compared the result between age groups.

**Figure 4:**
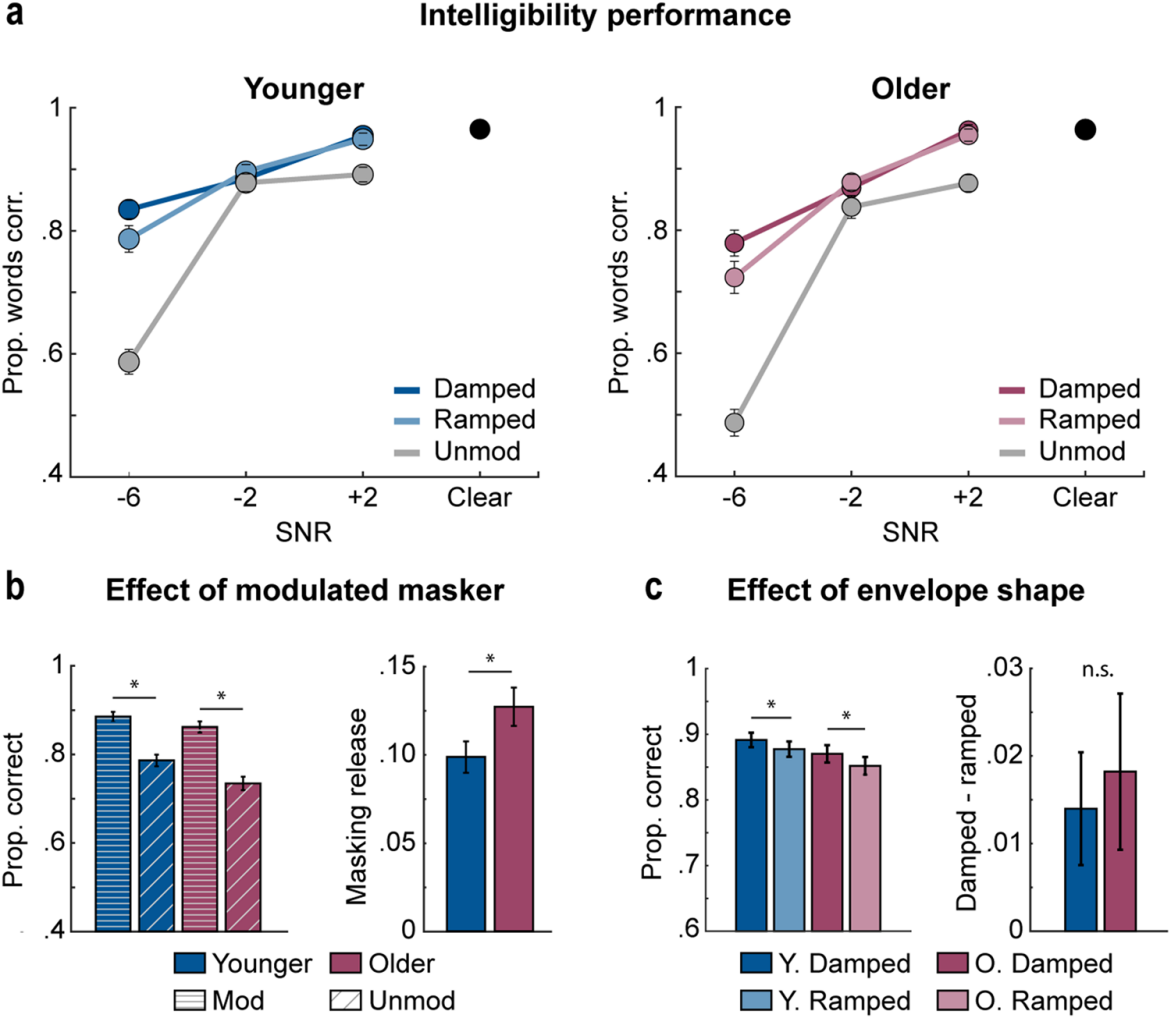
Intelligibility results for Experiment 2. (**a**) Mean proportion of correctly reported words are plotted as a function of SNR (− 6, − 2, + 2 dB, Clear [black dot]) for the different envelope conditions (damped, ramped, unmodulated) and for younger (left) and older adults (right). (**b**) Mean word report (left) is plotted for the different modulation types (modulated, unmodulated) and age groups (younger, older). The difference in performance between modulated and unmodulated maskers (masking release) is plotted for both age groups (right). Plots for modulated maskers reflect the mean across damped and ramped envelope shapes. (**c**) Mean word report (left) is plotted for the different masker envelope shapes (damped, ramped) and age groups (younger, older). The difference in performance between the damped and ramped envelope shapes is plotted for both age groups (right). Error bars reflect the standard error of the mean. *p < 0.05.

Average word report significantly declined with decreasing SNR [effect of SNR: *F*_2,272_ = 644.25, *p*_*GG*_ = 5.2 × 10^–80^, η^2^p = 0.83; Fig. 4a], and older adults exhibited worse overall performance compared to younger adults [effect of age group: *F*_1,136_ = 4.81, *p* = 0.03, η^2^p = 0.03]. We also found a significant SNR × age group interaction [*F*_2,272_ = 12.46, *p*_*GG*_ = 5.4 × 10^–5^ η^2^p = 0.08]. Follow up t-tests indicated age group differences at − 6 dB SNR [*t*_136_ = 2.98, *p*_*FDR*_ = 0.01, r_e_ = 0.25], but not at − 2 dB [*p*_*FDR*_ = 0.212] or + 2 dB [*p*_*FDR*_ = 0.968]. This shows that speech intelligibility during − 6 dB SNR was more challenging for older compared to younger subjects, while both groups performed equally well at − 2 and + 2 dB SNR.

We observed higher intelligibility for modulated compared to unmodulated maskers [effect of modulation type: *F*_1,136_ = 262.2, *p* = 2 × 10^–33^, η^2^p = 0.66, Fig. 4b left panel]. This release-from-masking effect (difference between modulated and unmodulated maskers) was greater for older compared to younger participants [modulation type × age group interaction: *F*_1,136_ = 4.14, *p* = 0.044, η^2^p  = 0.03; Fig. 4b right panel], although both groups showed significant release from masking [modulated vs. unmodulated: younger: *t*_67_ = 11.19, *p*_*FDR*_ = 6 × 10^–17^, r_e_ = 0.81; older: *t*_69_ = 11.83, *p*_*FDR*_ = 6 × 10^–18^, r_e_ = 0.82]. It appears that the modulated masker helped older adults to achieve a similar level of performance as younger adults [younger vs older adults for modulated masker: *p*_*FDR*_ = 0.162; Fig. 4b left panel], despite lower performance for the unmodulated masker [*t*_136_ = 2.57, *p*_*FDR*_ = 0.023, r_e_ = 0.21] (Fig. 4b right panel). This is not trivially due to a compressive effect at one or other extreme of performance: performance in the unmodulated and modulated conditions was off ceiling and floor for both age groups (Figs. 4a,b).

We also observed a modulation type × SNR interaction [*F*_2,272_ = 147.94, *p*_*GG*_ = 1.1 × 10^–36^, η^2^p = 0.52]. The difference between modulated and unmodulated performance (masking release) was larger at − 6 dB, compared both to − 2 dB [*t*_137_ = 15.99, *p*_*FDR*_ = 9.5 × 10^–33^, r_e_ = 0.81], and to + 2 dB [*t*_137_ = 10.96, *p*_*FDR*_ = 3 × 10^–20^, r_e_ = 0.68], although performance was enhanced for modulated compared to unmodulated maskers at all SNRs [− 6 dB: *t*_137_ = 9.19, *p*_*FDR*_ = 7.2 × 10^–16^, r_e_ = 0.62][− 2 dB: *t*_137_ = 2.49, *p*_*FDR*_ = 0.014, r_e_ = 0.21][+ 2 dB: *t*_137_ = 18.53, *p*_*FDR*_ = 2 × 10^–38^, r_e_ = 0.85]. The modulation type × SNR × age group interaction was not significant [*p* = 0.695].

Next, our analysis focused on the effects of masker envelope shape (damped, ramped) on speech intelligibility. Average word report declined with decreasing SNR [effect of SNR: *F*_2,272_ = 218.43, *p*_*GG*_ = 4.1 × 10^–42^, η^2^p  = 0.62; Fig. 4a]. We additionally observed a significant SNR × age group interaction [*F*_2,272_ = 8.004, *p*_*GG*_ = 0.002, η^2^p = 0.06], but did not find any significant effects during follow-up comparisons [*p*_*FDRs*_ > 0.07].

Consistent with Experiment 1, word report was higher when the envelope shape of the masker was damped compared to ramped [effect of envelope shape: *F*_1,136_ = 8.49, *p* = 0.004, η^2^p = 0.06; Fig. 4c left panel], and for both age groups [younger: *t*_67_ = 2.17, *p*_*FDR*_ = 0.045, r_e_ = 0.26; older: *t*_69_ = 2.04, *p*_*FDR*_ = 0.045, r_e_ = 0.24; envelope shape × age group interaction: *p* = 0.702; Fig. 4c]. Higher speech intelligibility for damped compared to ramped maskers was mainly driven by the most challenging SNR [6 dB: *t*_137_ = 3.5, *p*_*FDR*_ = 0.002, r_e_ = 0.29; envelope shape × SNR: *F*_2,272_ = 7.91, *p*_*GG*_ = 0.001, η^2^p  = 0.06; Fig. 4a], and not − 2 dB [*p*_*FDR*_ = 0.304], or + 2 dB [*p*_*FDR*_ = 0.304]. There were no other significant effects or interactions [*F* < 2, *p* > 0.16, η^2^p < 0.01].

Experiment 2 yielded two important findings. First, using engaging spoken stories, we show that older adults experience a larger speech intelligibility benefit from a modulated relative to an unmodulated masker compared to younger adults. This is in stark contrast to the results in Experiment 1 and the previous literature using short, disconnected sentences, which show a reduced intelligibility benefit in the presence of amplitude modulation for older compared to younger listeners^16,18–20,23,24,28,31,32,94^. Second, both older and younger participants exhibited better intelligibility when the babble masker was modulated with a damped compared to ramped envelope, partially replicating the results of Experiment 1, in which a benefit was seen for older, but not younger, adults. The shape of the modulated masker thus does not appear to strongly interact with age or the type of speech materials used during testing.

Experiments 1 and 2 differed substantially in speech materials and task procedure. In Experiment 3, we examine the effect of stimulus material and masker envelope on speech intelligibility. To ensure that narratives and isolated sentences are closely matched, we use target phrases/sentences either embedded in coherent stories or decontextualized in “scrambled” stories for which story sentences are shuffled in time. We use identical test phrases/sentences between the coherent and scrambled stories. As a result, we can more clearly determine whether removing the narrative arc of the story systematically alters the effects of age and masker envelope on speech intelligibility.

### Experiment 3: speech-intelligibility benefit for amplitude-modulated maskers depends on the speech materials in older adults

**Figure 5:**
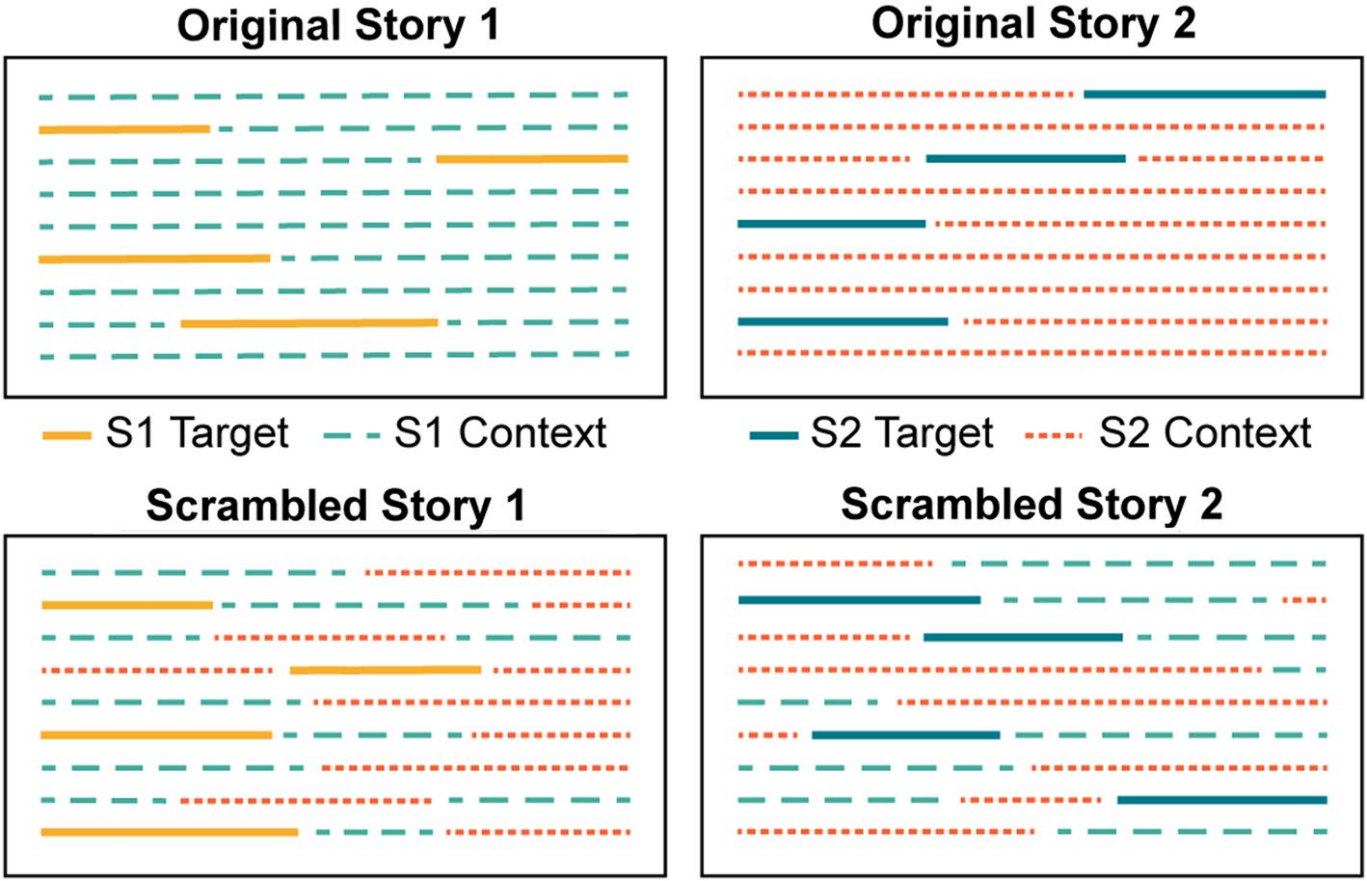
Stimulus schematic for Experiment 3. A schematic of the sentence structure of the original (top panel) and scrambled stories (bottom panel) is plotted. For the original stories, events were presented in their original order, whereas the scrambled stories contained the target phrases from one original story and a randomized mixture of the context sentences from both original stories. Target phrases/sentences used for intelligibility testing are plotted as solid lines (4 test sentences shown per story for simplicity), and context sentences are plotted as dashed lines. Note that the same target sentences are used in the original and scrambled stories.

Two 10-min stories (*Wave,* by D.M. Ouellet and *Alibi*, by Kristin Butcher) were selected and recorded for use in Experiment 3. These stories were written to be highly engaging but without complex language so that readers of any level may understand and enjoy the content. Two types of each story were created: original and scrambled. Original stories presented story events in the original order. Target phrases/sentences from the original stories were identified for intelligibility testing, as in Experiment 2 (Fig. 5, top panel). A scrambled story contained the same target phrases/sentences as one of the original stories and a randomized mixture of other (context) sentences drawn from both stories (Fig. 5, bottom panel). Scrambled stories thus lack a narrative thread, but are generated such that the test phrase/sentences used for intelligibility testing are identical across both story types.

Each story was masked by 12-talker babble noise, and the signal-to-noise ratio (− 6, − 2, + 2 dB, Clear) and amplitude envelope (unmodulated, 4-Hz modulated: damped, ramped) were manipulated. Younger (mean: 31.3 years; age-range: 21–38 years; 79 males 44 females) and older (mean: 63.2 years; age-range: 54–77 years; 44 males 77 females) adults without reported hearing or neurological issues listened to one of the four possible stories (2 original, 2 scrambled) and reported back cued sentences/phrases using the same online testing procedure as in Experiment 2 (Fig. 3). We calculated the proportion of correctly reported words for each story type (original, scrambled), envelope condition (damped, ramped, unmodulated) and SNR (− 6, − 2, + 2 dB, Clear) and compared the result between age groups.

**Figure 6:**
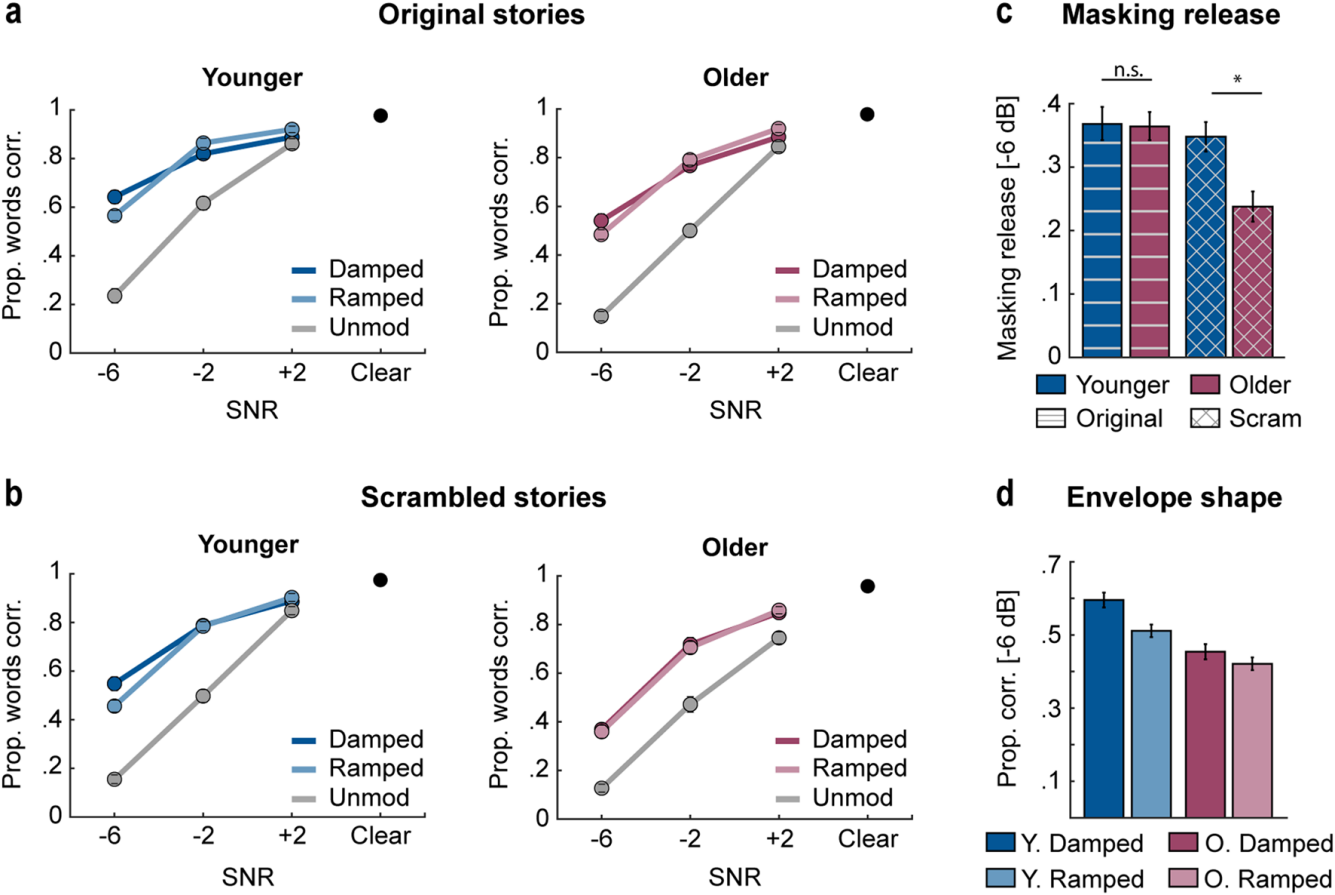
Intelligibility results for Experiment 3. (**a**) Mean proportion of correctly reported words for original stories is plotted as a function of SNR (– 6, − 2, + 2 dB, Clear [black dot]) for the different envelope conditions (damped, ramped, unmodulated) and for younger (left) and older adults (right). (**b**) Mean performance for scrambled stories is plotted as a function of SNR (− 6, − 2, + 2 dB, Clear [black dot]) for the different envelope conditions (damped, ramped, unmodulated) and for younger (left) and older adults (right). (**c**) Mean difference in intelligibility between modulated and unmodulated maskers (masking release) at − 6 dB SNR is plotted for each story type (original, scrambled) and age group (younger, older). (**d**) Mean performance at − 6 dB SNR is plotted for the different masker envelope shapes (damped, ramped) and age groups. Error bars reflect the standard error of the mean. *p < 0.05.

Consistent with Experiment 2, average word report declined with decreasing SNR [effect of SNR: *F*_2,480_ = 1665.46, *p* = 1.1 × 10^–216^, η^2^p  = 0.87; Fig. 6a,b]. Intelligibility was higher for original stories relative to scrambled [effect of story type: *F*_1,240_ = 20.12, *p* = 1.1 × 10^–5^, η^2^p  = 0.08], and higher for younger than older adults [effect of age group: *F*_1,240_ = 19.83, *p* = 1.3 × 10^–5^, η^2^p  = 0.08].

Speech intelligibility was also higher for modulated relative to unmodulated maskers [effect of modulation type: *F*_1,240_ = 999.81, *p* = 2 × 10^–87^, η^2^p  = 0.81; release-from-masking effect]. The modulation type × story type [*p* = 0.404], modulation type × age group [*p* = 0.698], and the modulation type × story type × age group [*p* = 0.051] interactions were not significant. All remaining 2- and 3-way interactions were significant [*p*_*GG*_s < 0.05]. However, because the 4-way interaction was also significant [modulation type × SNR × story type × age group: *F*_2,480_ = 3.98, *p*_*GG*_ = 0.021, η^2^p  = 0.02], we analyze this 4-way interaction and do not discuss the 2- and 3-way interactions any further.

To explore the significant 4-way interaction, we first calculated difference scores between average intelligibility scores for modulated and unmodulated trials (masking release) for each participant. Using post-hoc t-tests, we examined the effect of age group and story type on masking release at each SNR level. This revealed that the 4-way interaction was driven by group differences at – 6 dB SNR. At this challenging SNR, masking release for scrambled stories was larger for younger compared older adults [*t*_120_ = 3.3, *p*_*FDR*_ = 0.008*,* r_e_ = 0.29, Fig. 6c], whereas older adults benefited as much as younger adults from a modulated relative to an unmodulated masker for original stories [*p*_*FDR*_ = 0.91]. No differences were observed as a function of age group and story type at − 2 dB SNR or + 2 dB SNR [*p*_*FDR*_s > 0.06].

One potential explanation of this finding is that the reduced release from masking for older adults was simply due to the poor signal quality at − 6 dB leading to fewer intelligible words, and thus, less available context specifically for the older subject group. However, this seems unlikely since performance in the unmodulated condition for scrambled stories at − 6 dB SNR was not different between younger and older listeners [avg. words reported: younger: 16%; older: 13%; *p*_*FDR*_ > 0.4; Fig. 6b left panel vs right panel]. It is therefore unlikely that the reduced release from masking exhibited by older individuals for scrambled stories is due to less available context as a result of lower intelligibility for this condition. Furthermore, within the older group, performance in the unmodulated condition at − 6 dB SNR did not differ between scrambled and original stories [*p*_*FDR*_ > 0.4 Fig. 6a right panel vs 6b right panel]; therefore, the presence of context in the original stories is not solely driving the increased masking release for older adults, as such an effect should lead to better performance for both modulated and unmodulated conditions when listening to original stories. We tentatively conclude that the presence of meaningful context and perhaps the engagement that it fosters is qualitatively changing the older adults’ ability to benefit from masker modulation.

Next, we investigated whether the temporal profile of the modulated masker (damped vs. ramped) affects speech intelligibility for different story types and age groups. As expected, intelligibility declined with decreasing SNR [*F*_2, 480_ = 1041.51, *p*_*GG*_ = 1.2 × 10^–168^, η^2^p  = 0.81; Fig. 6a,b]; and was higher for original compared to scrambled stories [*F*_1,240_ = 22.31, *p* = 4 × 10^–6^, η^2^p  = 0.09]. The difference between original and scrambled stories was largest when the SNR was most challenging [− 6 dB: 0.13; − 2 dB: 0.06; + 2 dB: 0.03] [SNR × story type interaction: *F*_2,480_ = 15.12, *p*_*GG*_ = 7 × 10^–7^, η^2^p  = 0.06]. Intelligibility was also higher for younger compared to older adults [*F*_1,240_ = 20.16, *p* = 1.1 × 10^–5^, η^2^p  = 0.08]. The difference between older and younger adults was primarily observed at − 6 dB [*t*_242_ = 4.92, *p*_*FDR*_ = 5 × 10^–6^*,* r_e_ = 0.3] and − 2 dB [*t*_242_ = 3.53, *p*_*FDR*_ = 0.0007*,* r_e_ = 0.22], but not + 2 dB [*p*_*FDR*_ = 0.08] [SNR × age group interaction: *F*_2,480_ = 13.64, *p*_*GG*_ = 3 × 10^–6^, η^2^p  = 0.05].

The interaction between envelope shape × SNR was significant [*F*_2,480_ = 13.37, *p*_*GG*_ = 5 × 10^–6^, η^2^p  = 0.05]. Follow-up t-tests revealed that, at − 6 dB, target phrases/sentences were more intelligible when the masker was damped compared to ramped [*t*_243_ = 4.46, *p*_*FDR*_ = 4 × 10^–5^, r_e_ = 0.28]. At − 2 dB there was no effect of envelope shape [*p*_*FDR*_ = 0.276], while at + 2 dB [*t*_243_ = − 2.26, *p*_*FDR*_ = 0.04, r_e_ = 0.14] target phrases were more intelligible if the masker envelope was ramped, compared to damped. No other effects or interactions were significant [*F* < 2.5, *p* > 0.14, η^2^p  < 0.008].

To summarize Experiment 3, we demonstrate that the effect of age on the benefit of a fluctuating, compared to steady-state, masker critically depends on the speech material. Older adults experience less masking release than younger adults when listening to a stream of randomized sentences (scrambled story) that are similar to those commonly used in experimental aging research (cf. Experiment 1; see also^16,18–21,23,24,27–31,35,95^). However, for continuous speech with a topical thread, older adults benefit as much from a fluctuating masker as younger adults. These results suggest that research using disconnected sentences may systematically underestimate the speech-listening capabilities of older adults. Additionally, intelligibility is generally better when the babble masker had a damped compared to ramped temporal profile, effectively replicating Experiments 1 and 2.

## General discussion

In the current study, we investigated how intelligibility of masked speech in younger and older adults is affected by the nature of speech materials (isolated sentences vs engaging stories) and by the temporal profile of the masker’s amplitude envelope. We asked two specific questions: (1) Does the known age-related reduction in the speech-intelligibility benefit for modulated compared to unmodulated maskers depend on the nature of the speech materials? (2) Does speech intelligibility differ for modulated maskers with different temporal profiles (damped: sharp attack and gradual decay; ramped: gradual attack and fast decay), and does this differ between younger and older adults? We observed a reduced speech-intelligibility benefit for modulated over unmodulated background maskers in older relative to younger adults when individuals listened to disconnected sentences (Experiments 1 and 3). In marked contrast, older adults benefited more than younger adults (Experiment 2) or equally as much (Experiment 3) when they listened to engaging stories. We also generally observed better speech intelligibility for maskers with damped compared to ramped envelope shapes, suggesting that temporal profiles characterized by fast onsets and slow offsets may benefit intelligibility similarly across age groups and speech materials. Our results suggest that the well-documented deficit in ‘dip listening’ in older adults^16,18–21,23,24,27–31,35,95^ can be mitigated if the speech materials are engaging and contextually rich. Standard laboratory listening paradigms, utilizing disconnected sentences, elicit listening behavior that is qualitatively different from that observed when richer, continuous speech stimuli are used.

### Damped maskers interfere less with speech intelligibility than ramped maskers

The current study investigated whether the envelope shape of the masker (damped vs. ramped) influences the intelligibility of target speech. This research question was motivated by recent electrophysiological work in rodents and human participants^13,37–39^. Neural activity appears to synchronize more strongly with ramped than damped envelopes in younger people, and with damped compared to ramped envelopes in older people^13,37^. Furthermore, increased neural synchronization to a sound with a low-frequency amplitude modulation (e.g., ~ 4 Hz)^40,96,97^ may specifically predict declines in speech intelligibility when masked by a modulated background sound^38,39^. Based on these electrophysiological studies, we expected to observe reduced speech intelligibility for damped envelope shapes in older adults, and reduced intelligibility for ramped envelope shapes in younger adults. In contrast to our predictions, we generally observed better speech intelligibility for maskers with damped compared to ramped envelope shapes in both age groups, particularly when the SNR was low. Further, we did not find evidence that the effect of envelope shape differs between disconnected sentences and engaging stories (Experiment 3; Fig. 6). However, while the predictable masker rate of 4 Hz was motivated by electrophysiological work, we recognize that real-world listening situations do not typically have background maskers with predictable envelopes. Future studies could include a more ecologically valid manipulation of the amplitude envelope, such as using the temporal envelope of natural speech with salient ramped and damped envelopes by virtue of using words with those specific envelope shapes.

### Release from masking is not reduced in older compared to younger adults for engaging stories

Previous research indicates that aging is associated with a decline in processing temporal sound features^7–10,98–101^, and that temporal processing deficits may contribute to older adults experiencing difficulty understanding speech when background noise is present^11,12,17,100^. The persistent finding that older adults demonstrate either no benefit or a reduced speech-intelligibility benefit from a fluctuating relative to a flat envelope background masking sound^16,19,24,27,28^ has long been discussed as a prime example of temporal deficits limiting the ability of older adults to ‘glimpse’ target speech. In Experiments 1 and 3, we replicated previous findings that older adults benefit less from speech ‘glimpses’ compared to younger adults (Figs. 2b and 6c).

Critically, we also demonstrate that the ability to benefit from speech ‘glimpsing’ is only reduced in older adults when speech materials lack an overarching and engaging narrative context. When listening to engaging spoken stories, older adults demonstrated similar (Experiment 3; Fig. 6c) or even greater (Experiment 2; Fig. 4b) masking release compared to younger adults. Further, the interaction between age and speech material does not appear to have been driven by a ceiling effect in the younger participant group because we observed it at the most difficult SNR (− 6 dB) where performance was markedly lower than ceiling (Fig. 6). Our experiments demonstrate that the reduction in ‘speech glimpsing’ previously observed in older people may be specific to the speech materials commonly used in research studies, and may not generalize to listening situations with rich narrative structure.

Researchers have long concluded that the lack of benefit from speech ‘glimpses’ in older compared to younger individuals is due to increased spectrotemporal overlap between the target and masking signals in the auditory periphery (“energetic masking”), as a result of age-related hearing loss. Despite self-reports indicating the absence of hearing issues, our supplementary analysis (see Supplemental Document) indicates that our older adult sample may have slightly elevated hearing thresholds compared to younger adults (as would be expected). Elevated thresholds should be associated with reduced speech intelligibility and reduced release from masking overall, regardless of the sound type. Instead, our results suggest that the lack of benefit from speech ‘glimpses’ for isolated sentences might be related to other, perhaps more cognitive factors.

Several factors potentially contribute to the observed interaction between age and the type of materials (sentences vs stories) on release from masking. One critical difference is the higher degree of semantic context present in stories compared to disconnected sentences. Semantic context is well known to facilitate comprehension of words in disconnected sentences masked with noise for both older and younger adults^17,59–61,102–105^ and can alleviate listening effort for individuals with hearing impairment^106^. Moreover, spoken stories, such as the ones used in the current study, have an overarching topical thread that engages listeners^14^, and encourages them to continuously generate, update, and integrate story events and characters into a mental model that supports ongoing attention to the story^107–109^. This may recruit additional cognitive processes, compared to those recruited to understand isolated, unrelated sentences. The overarching narrative provides additional topic context that may enhance intelligibility, enabling participants to fill in missing information that was lost due to low SNR. Yet, context effects are unlikely to solely account for the older adults’ recovery of release from masking when listening to original stories. If this were due entirely to context effects, the added context of the original over scrambled stories should have led to better performance for the unmodulated original compared to scrambled story, and this was not observed.

Engaging spoken stories and disconnected sentences may also elicit different levels of motivation to listen. Motivation is crucial for the recruitment of cognitive resources during challenging tasks. A person will only invest cognitively if the activity is expected to be rewarding relative to the anticipated mental costs^64–66,110^. ‘Reward’ can take many forms and can be either extrinsic; for example, monetary rewards^111^ or intrinsic; through enjoyment and interest^71,112^. Spoken stories of the kind used here have been shown to be highly enjoyable and absorbing^14^ and elicit synchronized brain activity across listeners^90^, indicating their highly engaging nature. A recent study reported that listeners find stories as enjoyable and absorbing when they are masked by moderate background noise as when they are heard clearly, despite missing some words and finding listening more effortful in the former condition^14^. We speculate that older adults in the current study may have benefited from a modulated masker during story listening as much as younger adults because they enjoyed the story content, and were intrinsically motivated to invest additional cognitive resources to listen. We did not implement a measure of motivation or enjoyment following story listening, so it is not possible to relate motivation/enjoyment directly to intelligibility here. However, this interpretation is consistent with previous observations that older adults tend to engage less when tasks are less personally meaningful to them, perhaps in order to conserve mental resources^84,85^. Our results certainly point to large qualitative differences in listening behaviors for engaging spoken stories, compared to the disconnected sentence materials that are typically used in clinical and laboratory settings. We suggest that typical research approaches with disconnected sentences may underestimate the speech-listening abilities of older adults, especially in listening situations with narrated elements.

## Conclusions

Speech masked by a background sound with fluctuating amplitude is typically better understood than speech masked by sound with a relatively steady amplitude, but older adults have frequently been shown to benefit less from fluctuating maskers. This apparent deficit in the ability of older adults to ‘listen in the dips’ has been taken as a prime example of decreased temporal processing or reduced audibility in older individuals. Yet, speech intelligibility and masking release are typically investigated using short, disconnected sentences. Our results show that the release from masking depends on whether listeners are attending to disconnected sentences or to an engaging, connected, narrative. We replicated previous work showing a deficit in the speech-intelligibility benefit from amplitude fluctuations in older adults when they listened to disconnected sentences (Experiments 1 and 3). However, we further show that older adults either benefit more (Experiment 2) or similarly (Experiment 3) from modulated maskers relative to younger adults when listening to engaging spoken stories that follow a topical thread. Maskers with a damped temporal profile generally facilitated intelligibility and did not reliably interact with age or the type of speech material. Taken together, our data suggest that reduced ‘dip listening’ previously observed in older adults does not appear to generalize to engaging spoken stories. This result highlights that at least some deficits considered to be audiological may be more related to cognitive or motivational factors, and that the nature of the listening materials qualitatively changes listening behavior. Standard laboratory listening paradigms using disconnected sentences may underestimate the speech abilities of older adults.

## Materials and methods

### Experiment 1

#### Participants

One hundred and thirty-seven individuals (mean: 47.7 years; age-range: 18–71 years; 66 males 70 females 1 non-binary) without self-reported hearing loss, neurological issues, or psychiatric disorders participated in Experiment 1. Participants below age 50 were considered part of the ‘younger’ group (mean: 35.9 years; age-range: 18–49 years; 39 males, 29 females, 1 non-binary) and the remaining participants aged 50 and older were considered part of the ‘older’ group (mean: 59.6 years; age-range: 50–71 years; 31 males, 37 females). Participants were recruited from the Amazon Mechanical Turk online participant pool (MTurk; https://www.mturk.com/) via the participant sourcing platform Cloud Research (previously TurkPrime^86^). All participants provided informed consent prior to participation. The study was conducted in accordance with the Declaration of Helsinki, the Canadian Tri-Council Policy Statement on Ethical Conduct for Research Involving Humans (TCPS2-2018), and approved by the local Nonmedical Research Ethics Board of the University of Western Ontario (REB #112574).

Each individual received financial compensation of $5 USD following completion of the study ($10 hourly rate). Twenty-seven additional individuals participated in the study but were not included either due to reporting a technical error during data recording (N = 9), hearing aid usage or neurological issues (N = 7), not wearing headphones (N = 2), submitting the same one-word answers to all questions (N = 5), or scoring at floor (~ 10%) for all levels of background noise in the intelligibility task (N = 4). Online research can be subject to increased levels of random responders, since experimenters have less control over the testing environment compared to a laboratory setting. However, online studies have generally been shown to replicate findings of in-person data collection^113–118^ (see also Supplemental Document for the results of an in-lab pilot of Experiment 1), particularly if controls are in place to ensure compliance with study instructions.

#### Acoustic stimulation and procedure

All target sentences (N = 84) were spoken by the same female talker and ranged between 8 and 10 words in length (range of durations: 1.95–3.43 s). 12-talker babble noise from the Revised Speech in Noise test (R-SPIN)^119^ was added as a masker. Babble noise was either unmodulated (flat amplitude envelope) or amplitude modulated at a rate of 4 Hz with a damped (sharp attack and gradual decay) or ramped (gradual attack and sharp decay) envelope shape (Fig. 1). The modulation frequency of 4 Hz was chosen as it falls within the range of the low-frequency speech envelope^36,120^ and for consistency with previous electrophysiology work investigating how aging affects neural synchronization to the amplitude envelope^13,39,40,96^. Envelope shape was manipulated by varying parameters of the following equation:

1 $$\text{b } = {\text{t}}^{\text{z - 1}}\text{(1 }-\text{ t)}$$

where *t* is a time vector representing one cycle (0.250 s), *z* determines the envelope shape, and *b* is the resulting function used to modulate the noise. A *z* parameter of 2 generates a symmetrical envelope shape, while a value closer to 1 generates an envelope with a damped shape (sharp attack and gradual decay). Varying the *z* parameter also impacts the sharpness and half-life of each cycle. We used a *z* parameter of 1.15 to generate damped envelopes, each with a sharp onset and a 168.4 ms half-life^13,37^. Ramped envelopes (gradual attack and sharp decay) were created by mirroring the vector *b* (Fig. 1).

The signal-to-noise ratio (SNR) between the speech signal and the background babble was manipulated by adjusting the level of the sentence (target) relative to the babble masker (SNR levels: − 10, − 8, − 6, − 4, − 2, 0, + 2 dB). There were 21 possible stimulus conditions (7 SNRs × 3 envelope conditions = 21 stimulus conditions) that were tested in each block of trials (21 envelope conditions × 4 blocks = 84 total trials). To ensure intelligibility results were not confounded by specific sentences, 21 counterbalanced versions were generated, such that each sentence was heard with every SNR and envelope combination across versions. All sentence/babble mixtures were normalized relative to the same root-mean square amplitude (RMS).

The experiment was conducted online, using custom written JavaScript/html and jsPsych code (Version 6.1.0, a high-level JavaScript library used for precise stimulus control^121^). The experiment code was stored at an online repository (https://gitlab.pavlovia.org) and hosted via Pavlovia (https://pavlovia.org/). A test version was randomly assigned to each participant when data files were loaded into the internet browser. Prior to the main experimental procedures, participants were instructed to use headphones and complete the tasks in a quiet room free from distractions. We did not provide specifications as to the type/brand of equipment participants should use (e.g., computer, headphone type), but took steps to ensure participants complied with the instruction to use headphones (see “Online research quality assurance measures”).

During the main task (intelligibility task), participants were instructed to listen to each sentence and, after the sentence ended, type the words that they heard into a text box. Participants had unlimited time to type each response. Once participants submitted an answer, the next sentence would begin following a brief inter-trial silent interval of 0.25 s. Participants had the opportunity to take a break after each experimental block. The total duration of the intelligibility test was therefore dependent on the typing speed and total break length for each individual, but the intelligibility test duration typically ranged between 20 to 25 min.

#### Online research quality assurance measures

Participants completed two initial listening tasks at the beginning of the online session. First, participants listened to a 15-s stream of pink noise normalized to the same RMS amplitude as the sentences and were instructed to adjust their volume to a comfortable listening level. Participants had the option to replay the noise if they needed additional time to adjust their volume. This task ensured that participants could adjust their volume to a comfortable level prior to the intelligibility task, after which they were instructed to not make further adjustments.

Wearing headphones during the main experiment (intelligibility task) is an important condition of participation, since it can limit the influence of nearby distractions and help preserve stimulus characteristics, such as signal-to-noise ratio. In addition to explicitly asking participants whether they complied with instructions to wear headphones, participants also completed a headphone-check procedure (second listening task) to determine whether they were wearing headphones^122^. During the headphone-check procedure, participants performed a tone discrimination task (6 trials; ~ 2 min total duration), in which they determined which of three consecutive 200-Hz sine tones was the quietest. The three tones differed such that one was presented at the comfortable listening level, one at – 6 dB relative to the other two tones, and one at the comfortable listening level with a 180° phase difference between the left and right headphone channels (anti-phase tone). This task is straightforward over headphones, but difficult over loudspeakers, because the pressure waves generated from an anti-phase tone interfere^122^. If they were listening through loudspeakers, they would likely erroneously select the anti-phase tone as the quietest tone. This task provides another metric (in addition to self-report of headphone use), that could be used to flag participants who may not have been complying with instructions. No participants were excluded solely on the basis of performance on this test. Participants were excluded if they explicitly reported not wearing headphones during the task (N = 2).

#### Statistical analysis

Statistical analyses were conducted using IBM SPSS Statistics (version 27) for Windows and MATLAB (version 2018a). Details of the specific variables and statistical tests can be found in analysis subsections for each measure. The false-positive rate for multiple comparisons was controlled using false discovery rate (FDR)^123^. FDR corrected p-values are reported as *p*_*FDR*_*.* Effect sizes are reported as partial eta squared (η^2^p) for rmANOVAs and r_equivalent_ (r_e_)^124^, for *t*-tests. Greenhouse–Geisser corrected p-values are reported when sphericity assumptions have not been met (reported as *p*_*GG*_). This experiment was not preregistered. Data are available at the project website on the Open Science Framework (OSF: https://osf.io/swy57/). All figures were generated by the authors using MATLAB and Adobe Illustrator (version 2019).

#### Assessment of intelligibility

We calculated the proportion of correctly reported words for each SNR (− 10, − 8, − 6, − 4, − 2, 0, + 2 dB) and envelope condition (unmodulated, damped, ramped). Different or omitted words were counted as errors, but minor misspellings and incorrect grammatical number (singular vs. plural) were not. A logistic function was fit to the proportion of correctly reported words using the following equation:

2 $$\text{y = }\frac{K}{\left(1+{e}^{-\mathrm{r}\left(x- {x}_{o}\right)}\right)}$$

where *K* sets the curves maximum value, *r* is the slope, *x*_*0*_ is the inflection point or the speech reception threshold associated with 50% proportion of correct words, and *x* refers to the SNR values (− 10, − 8, − 6, − 4, − 2, 0, + 2 dB). We analyzed two parameters from each fit, the threshold and slope.

To examine differences in masking release as a function of age, we calculated the threshold and slope from the logistic function fit, separately for modulated (averaged across damped and ramped) and unmodulated trials. Threshold and slope were analyzed in separate mixed design repeated-measures analyses of variance (rmANOVAs) with modulation type (modulated, unmodulated) as a within-subject factor and age group (younger, older) as a between-subjects factor.

To analyze differences in speech intelligibility due to envelope shape (damped, ramped), thresholds and slopes from the logistic function fits were analyzed in separate rmANOVAs with envelope shape (damped, ramped) as a within-subjects factor and age group (younger, older) as a between-subjects factor.

### Experiment 2

#### Participants

One hundred and thirty-eight younger (mean: 30.1 years; age-range: 19–39 years; 37 males, 30 females) and older individuals (mean: 64.4 years; age-range: 53–80 years; 29 males, 41 females) without self-reported hearing loss, neurological issues, or psychiatric disorders participated in Experiment 2. All participants were recruited using identical procedures to Experiment 1, except that individuals who participated in Experiment 1 were precluded from participating in Experiment 2. Each individual received financial compensation of $6 USD following completion of the study ($10 hourly rate). Twenty-three additional individuals participated in the study but were not included either due to reporting a technical error during data recording (N = 6), hearing aid usage or neurological issues (N = 5), not wearing headphones (N = 4), identifying as a non-native English speaker (N = 2), or scoring ~ 50% or below on the intelligibility task when there was no masker (i.e., during clear speech; N = 6), suggesting participants were not attending during the task.

#### Acoustic stimulation and procedure

Acoustic stimulation and task procedures were adapted from a task developed previously^90^. One story (male talker) from “The Moth” story-telling podcast (https://themoth.org) was used as the target speech (*Reach for the Stars One Small Step at a Time*; by Richard Garriott, ~ 13 min). The target story had 12-talker babble noise added as a masker (R-SPIN)^119^. Babble noise could either be unmodulated (flat amplitude envelope) or amplitude modulated at a rate of 4 Hz with a ramped (gradual rise and sharp fall) or damped (sharp rise and gradual fall) envelope shape. Envelope shape was altered using identical parameters to Experiment 1 [cf. Equation (1)]. The signal-to-noise ratio (SNR) was manipulated by adjusting the dB level of both the story and masker. There were 3 possible envelope conditions (unmodulated, damped, ramped) and 3 different SNRs (− 6, − 2, + 2 dB SNR) along with a condition in which no masker was heard (clear), resulting in 10 total possible stimulus conditions (3 envelopes × 3 SNRs + clear = 10 conditions). Stimulus condition was pseudo-randomly varied approximately every 16 s (see Fig. 3) throughout each story. The length of the 16-s time window was determined by dividing the total duration of the story (in seconds) by the total number of trials. Each of the 10 stimulus conditions (3 envelopes × 3 SNRs + clear) were heard a total of 5 times over the course of the story (16 s × 10 conditions × 5 repetitions =  ~ 13 min). Three versions of condition order were generated to ensure that specific parts of the story were not confounded with a particular SNR and envelope combination. Within each version, SNR and envelope shape was varied pseudo-randomly such that a particular combination of SNR and envelope shape could not be heard twice in succession.

Phrases/sentences ranging from 4 to 7 words (range of durations: 0.85–2.6 s) were selected from the target story for intelligibility testing. These test phrases/sentences did not occur during the transition period from one SNR to the next (for approximately 1-s before and after the SNR transition). Two phrases/sentences per 16-s segment were selected, resulting in 100 possible test phrases for the target story (10 conditions × 5 repetitions × 2 phrases/sentences). One of the two selected phrases/sentences per 16-s segment was assigned to one intelligibility test set, whereas the other selected phrase/sentence was assigned to a second intelligibility test set (50 phrases/sentences per set). Having two test sets ensured that any observed intelligibility effects were not confounded by item (specific phrases/sentences) effects.

The experiment was conducted online using custom written JavaScript/html and jsPsych code hosted via Pavlovia (https://pavlovia.org/). During the main experiment, each participant listened to the target story and completed the intelligibility task. The condition order and intelligibility test set were randomly assigned to participants at the beginning of the experiment. Participants were instructed to use headphones and complete the tasks in a quiet room free from distractions. During story listening, a black fixation cross was presented at the center of the screen throughout the story. The fixation cross turned yellow two seconds prior to the beginning of a test phrase/sentence, cueing the participant to prepare for intelligibility testing (see Fig. 3). The fixation cross then turned green for the duration of the test phrase in the story, indicating to the participant the phrase they would be asked to report back. The story stopped with the offset of the test phrase, and an input text box appeared on the screen. Participants were asked to type their answer into the text box (no time limit), after which the story resumed from the beginning of the sentence most recently heard (allowing for story continuation). The total duration of the intelligibility task ranged between 25 to 30 min.

In order to familiarize participants with the intelligibility task, a brief practice block was presented prior to the main experiment. Participants heard a ~ 3-min story (a shortened version of *A Shoulder Bag to Cry On* by Laura Zimmerman), without added babble noise, and performed 12 trials of the intelligibility task (2 trials per 30-s segment, practice duration: ~ 5 min).

#### Online research quality assurance measures

Participants completed two initial listening tasks at the very beginning of the online session, as in Experiment 1. These preliminary tasks were meant to give the participant an opportunity to adjust their volume to a comfortable listening level and to provide a metric, aside from self-report, which could flag participants who may not be complying with instructions to wear headphones (headphone check). No participants were excluded solely on the basis of performance on this test, but were automatically excluded if they explicitly reported not wearing headphones during the task (N = 4). These tasks are described in Experiment 1.

#### Assessment of intelligibility

We calculated the proportion of correctly reported words for each envelope condition (damped, ramped, unmodulated) and SNR (− 6, − 2, + 2 dB, Clear) across the three versions of the target story. Different or omitted words were counted as errors, but minor misspellings, and incorrect grammatical number (singular vs. plural) were not. Contractions were also accepted as correct when the target contained the written out form of the contraction.

To analyze differences in masking release between age groups, mean performance for modulated (averaged across damped and ramped) and unmodulated trials were calculated and submitted to an rmANOVA with modulation type (modulated, unmodulated) and SNR (− 6, − 2, + 2 dB) as within-subject factors and age group (younger, older) as the between-subjects factor.

To examine the effect of envelope shape (damped, ramped) mean performance for damped and ramped trials were calculated and submitted to an rmANOVA with envelope shape (damped, ramped) and SNR (− 6, − 2, + 2 dB) as within-subject factors and age group (younger, older) as a between-subjects factor.

### Experiment 3

#### Participants

Two hundred and forty-four younger (mean: 31.3 years; age-range: 21–38 years; 79 males 44 females) and older individuals (mean: 63.2 years; age-range: 54–77 years; 44 males 77 females) without self-reported hearing loss, neurological issues, or psychiatric disorders participated in Experiment 3. Note that a higher number of participants were recruited for Experiment 3 than Experiments 1 and 2, because of the additional experimental factor: speech material type. All participants were recruited using identical procedures to Experiment 1 and 2, except that individuals who participated in Experiment 1 or 2 were precluded from participating in Experiment 3. Each individual received financial compensation of $5 USD following completion of the study ($10 hourly rate). Thirty-seven additional individuals participated in the study but were not included either due to reporting a technical error during data recording (N = 15), neurological issues (N = 7), not wearing headphones (N = 9), submitting random one-word answers to all questions (N = 3), or scoring ~ 50% or below on the intelligibility task when there was no masker (i.e., for clear speech; N = 3), suggesting participants were not attending during the task.

#### Acoustic stimulation and procedure

Stories were adapted from the content of two books (Story 1: *Wave,* by D.M. Ouellet; Story 2: *Alibi*, by Kristin Butcher) that were written to be engaging while avoiding complex language so that readers of any level may understand and enjoy the content. Shortened versions of the original stories were created and recorded by a female talker (duration of each story: ~ 10 min). Target phrases for the word-report task were identified in each of the two stories, as in Experiment 2 (see Fig. 5, top panel: solid lines). These phrases/sentences ranged from 4 to 7 words in length (range of durations: 0.66–2.05 s). Two phrases in each 15-s segment of the story were selected, resulting in 80 possible test phrases for story 1 and 80 possible test phrases for story 2. One of the two selected phrases per 15-s segment were assigned to one intelligibility test set, whereas the other selected phrases/sentences were assigned to a second intelligibility test set. This resulted in 4 total intelligibility test sets (2 per story), each comprising 40 test phrases/sentences. Having two intelligibility test sets for each story ensured that any observed effects were not confounded by the effects of specific word report items.

Half of the listeners performed the intelligibility task with the test phrases/sentences naturally embedded in the stories in their original, coherent form. The other half performed the intelligibility task with the test phrases/sentences embedded in “scrambled stories”. Four scrambled stories (one for each story and intelligibility test set: 2 stories × 2 intelligibility test sets) were created by embedding target phrases in a randomized mixture of other sentences drawn from both stories (see Fig. 5, bottom panel), such that an equal proportion of materials from each of the two stories entered each scrambled story version. The scrambled story condition therefore serves as an approximation of listening to disconnected sentences (cf. Experiment 1), since shuffling and intermixing the sentences limits any contextual relation between the embedded target phrases and the filler/contextual materials. In this design, each listener heard and reported sentences from only one of eight possible story conditions (2 stories × 2 intelligibility test sets × 2 story type [original, scrambled]), and we measure word-report performance on exactly the same material when it is presented in an engaging story versus decontextualized as disjointed sentences.

Each original and scrambled story was masked by 12-talker babble noise (R-SPIN)^119^. The SNR (− 6, − 2, + 2 dB, clear), and envelope condition (ramped, damped, unmodulated) varied pseudo-randomly as in Experiment 2, with the exception that the stimulus condition changed every 15 s (instead of the 16 s period used in Experiment 2), since the stories used here were shorter in duration. Each of the 10 stimulus conditions (3 envelopes × 3 SNRs + clear) were heard four times over the course of the story (15 s × 10 conditions × 4 repetitions =  ~ 10 min). Three different stimulus condition orders were generated for each story to ensure that specific parts of a story were not confounded with a particular SNR and envelope combination. Within each version, SNR and envelope shape were varied pseudo-randomly such that a particular combination of SNR and envelope shape could not be heard twice in succession.

The experiment was conducted online using custom written JavaScript/html and jsPsych code hosted via Pavlovia (https://pavlovia.org/). Each participant was pseudo-randomly assigned to one of the 8 story conditions described (2 stories × 2 intelligibility test sets × story type [original, scrambled]) and to one of the three stimulus condition orders. Participants were instructed to use headphones and complete the tasks in a quiet room free from distractions. In the main experiment, the participant listened to a story and completed the same intelligibility task used in Experiment 2 (see Fig. 3). Participants had unlimited time to submit each response. The total duration of the intelligibility test ranged between 15 to 20 min. In order to familiarize participants with the intelligibility task, a brief practice block was presented prior to the main experiment. Participants heard a ~ 3-min story (a shortened version of *A Shoulder Bag to Cry On* by Laura Zimmerman), without added babble noise, and performed 12 trials of the intelligibility task (2 trials per 30-s segment, practice duration: ~ 5 min).

#### Online research quality assurance measures

As in Experiment 1 and 2, participants completed two initial listening tasks at the very beginning of the online session. These preliminary tasks were meant to give the participant an opportunity to adjust their volume to a comfortable listening level and to provide a metric, aside from self-report, which could flag participants who may not be complying with instructions to wear headphones (headphone check). No participants were excluded solely on the basis of performance on this test, but were automatically excluded if they explicitly reported not wearing headphones during the task (N = 9). Specific methods are described in Experiment 1.

#### Assessment of intelligibility

We calculated the proportion of correctly reported words for each envelope type (damped, ramped, unmodulated) and SNR condition (− 6, − 2, + 2 dB, Clear), separately for original and scrambled stories, and separately for each version of the word-report task for each story. Different or omitted words were counted as errors, but minor misspellings, and incorrect grammatical number (singular vs. plural) were not. Contractions were also accepted as correct when the target contained the written-out form of the contraction.

Effects of modulation type were tested using an ANOVA (within-subjects factors modulation type (modulated [averaged across ramped and damped], unmodulated) and SNR (− 6, − 2, + 2) and the between-subjects factors story type (original, scrambled) and age group (younger, older).

Effects of envelope shape were analyzed using an rmANOVA with the within-subjects factors envelope shape (damped, ramped) and SNR (− 6, − 2, + 2) and the between-subjects factors story type (unaltered, scrambled) and age group (younger, older).

## Supplementary Information

Supplementary Information.

## References

[1] Drullman R, Festen JM, Plomp R (1994). Effect of temporal envelope smearing on speech reception. J. Acoust. Soc. Am..

[2] Shannon RV, Zeng F-G, Kamath V, Wygonski J, Ekelid M (1995). Speech recognition with primarily temporal cues. Am. Assoc. Adv. Sci..

[3] van der Horst R, Leeuw AR, Dreschler WA (1999). Importance of temporal-envelope cues in consonant recognition. J. Acoust. Soc. Am..

[4] Festen JM, Plomp R (1990). Effects of fluctuating noise and interfering speech on the speech-reception threshold for impaired and normal hearing. J. Acoust. Soc. Am..

[5] Miller GA, Licklider JCR (1950). The intelligibility of interrupted speech. J. Acoust. Soc. Am..

[6] Cooke M (2006). A glimpsing model of speech perception in noise. J. Acoust. Soc. Am..

[7] Gordon-Salant S, Fitzgibbons PJ (1999). Profile of auditory temporal processing in older listeners. J. Speech Lang. Hear. Res..

[8] Ruggles D, Bharadwaj H, Shinn-Cunningham BG (2012). Why middle-aged listeners have trouble hearing in everyday settings. Curr. Biol..

[9] Grose JH, Mamo SK, Buss E, Hall JW (2015). Temporal processing deficits in middle age. Am. J. Audiol..

[10] Bharadwaj HM, Verhulst S, Shaheen L, Charles Liberman M, Shinn-Cunningham BG (2014). Cochlear neuropathy and the coding of supra-threshold sound. Front. Syst. Neurosci..

[11] Frisina DR, Frisina RD (1997). Speech recognition in noise and presbycusis: Relations to possible neural mechanisms. Hear. Res..

[12] Gordon-Salant S (2006). Speech perception and auditory temporal processing performance by older listeners: Implications for real-world communication. Semin. Hear..

[13] Irsik VC, Almanaseer A, Johnsrude IS, Herrmann B (2021). Cortical responses to the amplitude envelopes of sounds change with age. J. Neurosci..

[14] Herrmann B, Johnsrude IS (2020). Absorption and enjoyment during listening to acoustically masked stories. Trends Hear..

[15] Davis MH, Johnsrude IS (2003). Hierarchical processing in spoken language comprehension. J. Neurosci..

[16] Dubno JR, Horwitz AR, Ahlstrom JB (2002). Benefit of modulated maskers for speech recognition by younger and older adults with normal hearing. J. Acoust. Soc. Am..

[17] Pichora-Fuller MK, Schneider BA, Daneman M (1995). How young and old adults listen to and remember speech in noise. J. Acoust. Soc. Am..

[18] Stuart A, Phillips DP (1998). Deficits in auditory temporal resolution revealed by a comparison of word recognition under interrupted and continuous noise masking. Semin. Speech Lang..

[19] Summers V, Molis MR (2004). Speech recognition in fluctuating and continuous maskers: Effects of hearing loss and presentation level. J. Speech Lang. Hear. Res..

[20] Turner CW, Souza PE, Forget LN (1995). Use of temporal envelope cues in speech recognition by normal and hearing-impaired listeners. J. Acoust. Soc. Am..

[21] Moore BCJ (2008). The role of temporal fine structure processing in pitch perception, masking, and speech perception for normal-hearing and hearing-impaired people. J. Assoc. Res. Otolaryngol..

[22] Vestergaard MD, Fyson NRC, Patterson RD (2011). The mutual roles of temporal glimpsing and vocal characteristics in cocktail-party listening. J. Acoust. Soc. Am..

[23] Gustafsson HA, Arlinger SD (1994). Masking of speech by amplitude-modulated noise. J. Acoust. Soc. Am..

[24] Stuart A, Phillips DP, Green WB (1995). Word recognition performance in continuous and interrupted broad-band noise by normal hearing and simulated hearing-impaired listeners. Am. J. Otol..

[25] Füllgrabe C, Berthommier F, Lorenzi C (2006). Masking release for consonant features in temporally fluctuating background noise. Hear. Res..

[26] Gnansia D, Jourdes V, Lorenzi C (2008). Effect of masker modulation depth on speech masking release. Hear. Res..

[27] Bacon SP, Opie JM, Montoya DY (1998). The effects of hearing loss and noise masking on the masking release for speech in temporally complex backgrounds. J. Speech Lang. Hear. Res..

[28] George ELJ, Festen JM, Houtgast T (2006). Factors affecting masking release for speech in modulated noise for normal-hearing and hearing-impaired listeners. J. Acoust. Soc. Am..

[29] Lorenzi C, Husson M, Ardoint M, Debruille X (2006). Speech masking release in listeners with flat hearing loss: Effects of masker fluctuation rate on identification scores and phonetic feature reception. Int. J. Audiol..

[30] Lorenzi C, Moore BCJ (2008). Role of temporal envelope and fine structure cues in speech perception: A review. Proc. Int. Symp. Audit. Audiol. Res..

[31] Dubno JR, Horwitz AR, Ahlstrom JB (2003). Recovery from prior stimulation: Masking of speech by interrupted noise for younger and older adults with normal hearing. J. Acoust. Soc. Am..

[32] Eisenberg LS, Dirks DD, Bell TS (1995). Speech recognition in amplitude-modulated noise of listeners with normal and listeners with impaired hearing. J. Speech Hear. Res..

[33] Gilbert G, Bergeras I, Voillery D, Lorenzi C (2007). Effects of periodic interruptions on the intelligibility of speech based on temporal fine-structure or envelope cues. J. Acoust. Soc. Am..

[34] Gnansia D, Péan V, Meyer B, Lorenzi C (2009). Effects of spectral smearing and temporal fine structure degradation on speech masking release. J. Acoust. Soc. Am..

[35] Lorenzi C, Gilbert G, Carn H, Garnier S, Moore BCJ (2006). Speech perception problems of the hearing impaired reflect inability to use temporal fine structure. Proc. Natl. Acad. Sci. USA..

[36] Rosen S (1992). Temporal information in speech: Acoustic, auditory and linguistic aspects. Philos. Trans. R. Soc. Lond. B..

[37] Herrmann B, Parthasarathy A, Bartlett EL (2017). Ageing affects dual encoding of periodicity and envelope shape in rat inferior colliculus neurons. Eur. J. Neurosci..

[38] Millman RE, Mattys SL, Gouws AD, Prendergast G (2017). Magnified neural envelope coding predicts deficits in speech perception in noise. J. Neurosci..

[39] Goossens T, Vercammen C, Wouters J, van Wieringen A (2018). Neural envelope encoding predicts speech perception performance for normal-hearing and hearing-impaired adults. Hear. Res..

[40] Goossens T, Vercammen C, Wouters J, van Wieringen A (2016). Aging affects neural synchronization to speech-related acoustic modulations. Front. Aging Neurosci..

[41] Moore BCJ, Glasberg BR (1993). Simulation of the effects of loudness recruitment and threshold elevation on the intelligibility of speech in quiet and in a background of speech. J. Acoust. Soc. Am..

[42] Schlittenlacher J, Moore BCJ (2016). Discrimination of amplitude-modulation depth by subjects with normal and impaired hearing. J. Acoust. Soc. Am..

[43] Henry KS, Kale S, Heinz MG (2014). Noise-induced hearing loss increases the temporal precision of complex envelope coding by auditory-nerve fibers. Front. Syst. Neurosci..

[44] Kale S, Heinz MG (2010). Envelope coding in auditory nerve fibers following noise-induced hearing loss. J. Assoc. Res. Otolaryngol..

[45] Zhong Z, Henry KS, Heinz MG (2014). Sensorineural hearing loss amplifies neural coding of envelope information in the central auditory system of chinchillas. Hear. Res..

[46] Schiffrin D (1984). How a story says what it means and does. Text Interdiscip. J. Study Discourse.

[47] Jefferson G (1978). Sequential aspects of storytelling in conversation. Stud. Org. Convers. Interact..

[48] Ochs E, Taylor C (1992). Family narrative as political activity. Discourse Soc..

[49] Pasupathi M, Lucas S, Coombs A (2002). Conversational functions of autobiographical remembering: Long-married couples talk about conflicts and pleasant topics. Discourse Process..

[50] Ervin-Tripp, S. M. & Küntay, A. C. The occasioning and structure of conversational stories. in *Conversation: Cognitive, communicative and social perspectives* 133–166 (John Benjamins, 1997). doi:10.1075/tsl.34.06erv.

[51] Bohanek JG (2009). Narrative interaction in family dinnertime conversations. Merrill. Palmer. Q..

[52] Eisenberg AR (1985). Learning to describe past experiences in conversation. Discourse Process..

[53] Fivush R, Bohanek JG, Zaman W (2011). Personal and intergenerational narratives in relation to adolescents’ well-being. New Dir. Child Adolesc. Dev..

[54] McLean KC, Pasupathi M, Pals JL (2007). Selves creating stories creating selves: A process model of self-development. Personal. Soc. Psychol. Rev..

[55] Mullen MK, Yi S (1995). The cultural context of talk about the past: Implications for the development of autobiographical memory. Cogn. Dev..

[56] Ochs E, Capps L (1996). Narrating the self. Annu. Rev. Anthropol..

[57] Ochs E, Smith R, Taylor C (1989). Detective stories at dinnertime: Problem-solving through co-narration. Cult. Dyn..

[58] Ochs E, Taylor C, Rudolph D, Smith R (1992). Storytelling as a theory-building activity. Discourse Process..

[59] Kalikow DN, Stevens KN, Elliott LL (1977). Development of a test of speech intelligibility in noise using sentence materials with controlled word predictability. J. Acoust. Soc. Am..

[60] Dubno JR, Ahlstrom JB, Horwitz AR (2000). Use of context by young and aged adults with normal hearing. J. Acoust. Soc. Am..

[61] Nittrouer S, Boothroyd A (1990). Context effects in phoneme and word recognition by young children and older adults. J. Acoust. Soc. Am..

[62] Sheldon S, Pichora-Fuller MK, Schneider BA (2008). Priming and sentence context support listening to noise-vocoded speech by younger and older adults. J. Acoust. Soc. Am..

[63] Cohen G, Faulkner D (1983). Word recognition: Age differences in contextual facilitation effects. Br. J. Psychol..

[64] Botvinick M, Braver T (2015). Motivation and cognitive control: From behavior to neural mechanism. Annu. Rev. Psychol..

[65] Kool W, McGuire JT, Rosen ZB, Botvinick M (2010). Decision making and the avoidance of cognitive demand. J. Exp. Psychol. Gen..

[66] Yee DM, Braver TS (2018). Interactions of motivation and cognitive control. Curr. Opin. Behav. Sci..

[67] Herrmann B, Johnsrude IS (2020). A model of listening engagement (MoLE). Hear. Res..

[68] Eckert MA, Teubner-Rhodes S, Vaden KI (2016). Is listening in noise worth it? The neurobiology of speech recognition in challenging listening conditions. Ear Hear..

[69] Peelle JE (2018). Listening effort: How the cognitive consequences of acoustic challenge are reflected in brain and behavior. Ear Hear..

[70] Pichora-Fuller MK (2016). Hearing impairment and cognitive energy: The framework for understanding effortful listening (FUEL). Ear Hear..

[71] Matthen M (2016). Effort and displeasure in people who are hard of hearing. Ear Hear..

[72] Lalor EC, Foxe JJ (2010). Neural responses to uninterrupted natural speech can be extracted with precise temporal resolution. Eur. J. Neurosci..

[73] Ki JJ, Kelly SP, Parra LC (2016). Attention strongly modulates reliability of neural responses to naturalistic narrative stimuli. J. Neurosci..

[74] Polonenko MJ, Maddox RK (2021). Exposing distinct subcortical components of the auditory brainstem response evoked by continuous naturalistic speech. Elife.

[75] Schmälzle R, Häcker FEK, Honey CJ, Hasson U (2015). Engaged listeners: Shared neural processing of powerful political speeches. Soc. Cogn. Affect. Neurosci..

[76] Broderick MP, Anderson AJ, Di Liberto GM, Crosse MJ, Lalor EC (2018). Electrophysiological correlates of semantic dissimilarity reflect the comprehension of natural, narrative speech. Curr. Biol..

[77] Fiedler L, Wöstmann M, Herbst SK, Obleser J (2019). Late cortical tracking of ignored speech facilitates neural selectivity in acoustically challenging conditions. Neuroimage.

[78] Keitel A, Ince RAA, Gross J, Kayser C (2017). Auditory cortical delta-entrainment interacts with oscillatory power in multiple fronto-parietal networks. Neuroimage.

[79] Puvvada KC, Simon JZ (2017). Cortical representations of speech in a multitalker auditory scene. J. Neurosci..

[80] Brodbeck C, Jiao A, Hong LE, Simon JZ (2020). Neural speech restoration at the cocktail party: Auditory cortex recovers masked speech of both attended and ignored speakers. PLoS Biol..

[81] Broderick MP, Anderson AJ, Lalor EC (2019). Semantic context enhances the early auditory encoding of natural speech. J. Neurosci..

[82] Broderick MP, Di Liberto G, Anderson A, Rofes A, Lalor E (2020). Dissociable electrophysiological measures of natural language processing reveal differences in speech comprehension strategy in healthy ageing. Sci. Rep..

[83] Erb, J., Schmitt, L.M. & Obleser, J. Temporal selectivity declines in the aging human auditory cortex. *Elife* **9**, e55300 (2020). 10.7554/eLife.55300 PMC7410487 32618270

[84] Hess TM, Ennis GE (2014). Assessment of adult age differences in task engagement: The utility of systolic blood pressure. Motiv. Emot..

[85] Hess TM (2014). Selective engagement of cognitive resources. Perspect. Psychol. Sci..

[86] Litman, L., Robinson, J. & Abberbock, T. TurkPrime.com: A versatile crowdsourcing data acquisition platform for the behavioral sciences. *Behav. Res. Methods* **49**, 433–442 (2017). 10.3758/s13428-016-0727-z PMC5405057 27071389

[87] Smits C, Kapteyn TS, Houtgast T (2004). Development and validation of an automatic speech-in-noise screening test by telephone. Int. J. Audiol..

[88] Schneider BA, Daneman M, Murphy DR (2005). Speech comprehension difficulties in older adults: Cognitive slowing or age-related changes in hearing?. Psychol. Aging.

[89] Bernstein JGW, Brungart DS (2011). Effects of spectral smearing and temporal fine-structure distortion on the fluctuating-masker benefit for speech at a fixed signal-to-noise ratio. J. Acoust. Soc. Am..

[90] Irsik, V. C., Johnsrude, I. S. & Herrmann, B. Synchronized neural activity indexes engagement with spoken stories under acoustic masking. *bioRxiv* (2021).

[91] Dmochowski JP, Sajda P, Dias J, Parra LC (2012). Correlated components of ongoing EEG point to emotionally laden attention: A possible marker of engagement?. Front. Hum. Neurosci..

[92] Hasson U, Malach R, Heeger DJ (2010). Reliability of cortical activity during natural stimulation. Trends Cogn. Sci..

[93] Hasson, U., Nir, Y., Levy, I., Fuhrmann, G. & Malach, R. Intersubject synchronization of cortical activity during natural vision. *Science* **303**, 1634–1640 (2004). 10.1126/science.1089506 15016991

[94] Léger AC, Moore BCJ, Lorenzi C (2012). Temporal and spectral masking release in low- and mid-frequency regions for normal-hearing and hearing-impaired listeners. J. Acoust. Soc. Am..

[95] Peters RW, Moore BCJ, Baer T (1998). Speech reception thresholds in noise with and without spectral and temporal dips for hearing-impaired and normally hearing people. J. Acoust. Soc. Am..

[96] Goossens T, Vercammen C, Wouters J, van Wieringen A (2019). The association between hearing impairment and neural envelope encoding at different ages. Neurobiol. Aging.

[97] Herrmann B, Buckland C, Johnsrude IS (2019). Neural signatures of temporal regularity processing in sounds differ between younger and older adults. Neurobiol. Aging.

[98] Fitzgibbons PJ, Gordon-Salant S (1995). Age effects on duration discrimination with simple and complex stimuli. J. Acoust. Soc. Am..

[99] Fitzgibbons, P. J. & Gordon-Salant, S. Auditory temporal order perception in younger and older adults. *J. Speech, Lang. Hear. Res.* **41**, 1052–1060 (1998). 10.1044/jslhr.4105.1052 9771628

[100] Pichora-Fuller MK, Schneider BA, MacDonald E, Pass HE, Brown S (2007). Temporal jitter disrupts speech intelligibility: A simulation of auditory aging. Hear. Res..

[101] Schneider BA, Pichora-Fuller MK (2001). Age-related changes in temporal processing: Implications for speech perception. Semin. Hear..

[102] Obleser J, Wise RJS, Dresner MA, Scott SK (2007). Functional integration across brain regions improves speech perception under adverse listening conditions. J. Neurosci..

[103] Davis MH, Ford MA, Kherif F, Johnsrude IS (2011). Does semantic context benefit speech understanding through ‘top-down’ processes? Evidence from time-resolved sparse fMRI. J. Cogn. Neurosci..

[104] Miller GA, Heise GA, Lichten W (1951). The intelligibility of speech as a function of the context of the test materials. J. Exp. Psychol..

[105] Bashford JA, Riener KR, Warren RM (1992). Increasing the intelligibility of speech through multiple phonemic restorations. Percept. Psychophys..

[106] Holmes E, Folkeard P, Johnsrude IS, Scollie S (2018). Semantic context improves speech intelligibility and reduces listening effort for listeners with hearing impairment. Int. J. Audiol..

[107] Mar RA, Oatley K (2008). The function of fiction is the abstraction and simulation of social experience. Perspect. Psychol. Sci..

[108] Zwaan RA (2016). Situation models, mental simulations, and abstract concepts in discourse comprehension. Psychon. Bull. Rev..

[109] Zwaan RA, Langston MC, Graesser AC (1995). The construction of situation models in narrative comprehension: An event-indexing model. Psychol. Sci..

[110] Botvinick M, Huffstetler S, McGuire JT (2009). Effort discounting in human nucleus accumbens. Cogn. Affect. Behav. Neurosci..

[111] Bénabou R, Tirole J (2003). Intrinsic and extrinsic motivation. Rev. Econ. Stud..

[112] Ryan RM, Deci EL (2000). Intrinsic and extrinsic motivations: Classic definitions and new directions. Contemp. Educ. Psychol..

[113] Gosling SD, Vazire S, Srivastava S, John OP (2004). Should we trust web-based studies? A comparative analysis of six preconceptions about internet questionnaires. Am. Psychol..

[114] Buhrmester M, Kwang T, Gosling SD (2011). Amazon’s Mechanical Turk: A new source of inexpensive, yet high-quality, data?. Perspect. Psychol. Sci..

[115] Thomas KA, Clifford S (2017). Validity and Mechanical Turk: An assessment of exclusion methods and interactive experiments. Comput. Human Behav..

[116] Berinsky AJ, Margolis MF, Sances MW (2014). Separating the shirkers from the workers? Making sure respondents pay attention on self-administered surveys. Am. J. Pol. Sci..

[117] Buchanan EM, Scofield JE (2018). Methods to detect low quality data and its implication for psychological research. Behav. Res. Methods.

[118] Mason W, Suri S (2012). Conducting behavioral research on Amazon’s Mechanical Turk. Behav. Res. Methods.

[119] Bilger, R. C. *Manual for the clinical use of the Revised SPIN test*. (University of Illinois Press, London, 1984).

[120] Edwards E, Chang EF (2013). Syllabic (~2-5Hz) and fluctuation (~1-10Hz) ranges in speech and auditory processing. Hear. Res..

[121] de Leeuw JR (2015). jsPsych: A JavaScript library for creating behavioral experiments in a Web browser. Behav. Res. Methods.

[122] Woods KJP, Siegel MH, Traer J, McDermott JH (2017). Headphone screening to facilitate web-based auditory experiments. Attent. Percept. Psychophys..

[123] Benjamini Y, Hochberg Y (1995). Controlling the false discovery rate: A practical and powerful approach to multiple testing. J. R. Stat. Soc. Ser. B.

[124] Rosenthal R, Rubin DB (2003). r equivalent: A simple effect size indicator. Psychol. Methods.

