# Supplementary material for: Age-related deficits in dip-listening evident for isolated sentences but not for spoken stories
Source: Sci Rep. 2022 Apr 7;12:5898. doi: 10.1038/s41598-022-09805-6 (PMC8991280; doi:10.1038/s41598-022-09805-6)
Supplement: Supplementary file 1 — Supplementary Information. [file 41598_2022_9805_MOESM1_ESM.docx]

**Supplementary Document:**

Age-related deficits in dip-listening evident for isolated sentences but not for spoken stories

Vanessa C. Irsik^1,*^, Ingrid S. Johnsrude^1,2^, and Björn Herrmann^1,3,4^

^1^ Department of Psychology & The Brain and Mind Institute,
The University of Western Ontario, London, ON, Canada N6A 3K7

^2^ School of Communication and Speech Disorders,
The University of Western Ontario, London, ON, N6A 5B7, Canada

^3^ Rotman Research Institute,
Baycrest, M6A 2E1, Toronto, ON, Canada

^4^ Department of Psychology,
University of Toronto, M5S 1A1, Toronto, ON, Canada

* Correspondence concerning this article should be addressed to Vanessa C. Irsik, The Brain and Mind Institute, The University of Western Ontario, London, Ontario, N6A 5B7, Canada. E-mail: virsik@uwo.ca

**Acknowledgements:**

This research was supported by the Canadian Institutes of Health Research (MOP133450 to I.S. Johnsrude). BH was supported by the Canada Research Chair program.

# Supplementary Methods and Results

## Effect of testing environment

In an effort to demonstrate the relative quality of data collected online compared to a more controlled laboratory setting, we report the results of a pilot version of Experiment 1 conducted in-lab. Twenty-eight younger adults (mean: 18.2 years; age-range: 17-20 years; 7 males 20 females 1 non-binary) without self-reported hearing loss, neurological issues, or psychiatric disorders were recruited for the in-lab pilot study. A sample of younger participants from Experiment 1 (mean: 21.8 years; age-range: 18-34 years; 17 males 9 females 1 non-binary) were selected for comparison with the pilot data. A participant from Experiment 1 was selected if they were less than thirty-five years old. It was not possible to further age match the Experiment 1 sample with the in-lab pilot without excessively reducing the sample size. Individuals participating in-lab (mainly 1^st^ year undergraduate students) were overall younger than those participating online (recruitment from the community).

The pilot experiment was conducted in a single-walled sound-attenuating booth (Eckel Industries). Sounds were delivered through Sennheiser (HD 25 Light) headphones, using a Steinberg UR22 external soundcard controlled by a PC (Windows 10) and Psychtoolbox (Version 3) in MATLAB (R2017b).

During the pilot experiment, participants were instructed to listen to isolated sentences with added babble noise and type the words they heard into a text box. The sentences, manipulation of SNR (SNR levels: -10, -8, -6, -4, -2, 0, +2 dB), and masker envelope (unmodulated, 4-Hz modulated: damped, ramped) were all identical to Experiment 1. There were 21 possible stimulus conditions (7 SNRs × 3 envelope conditions = 21 stimulus conditions) that were tested in each of the 4 blocks of trials (21 envelope conditions × 4 blocks = 84 total trials). A logistic function was fit to the proportion of correctly reported words for each envelope condition (damped, ramped, unmodulated; Figure S1a). We restricted our analysis to the threshold parameter from each fit, as no significant effects were observed for slope in Experiment 1.

To examine potential differences in SNR threshold between in-lab and online data, threshold values were analyzed in a mixed design repeated-measures analyses of variance (rmANOVA) with modulation type (damped, ramped, unmodulated) as a within-subject factor and testing environment (younger, older) as a between-subjects factor.


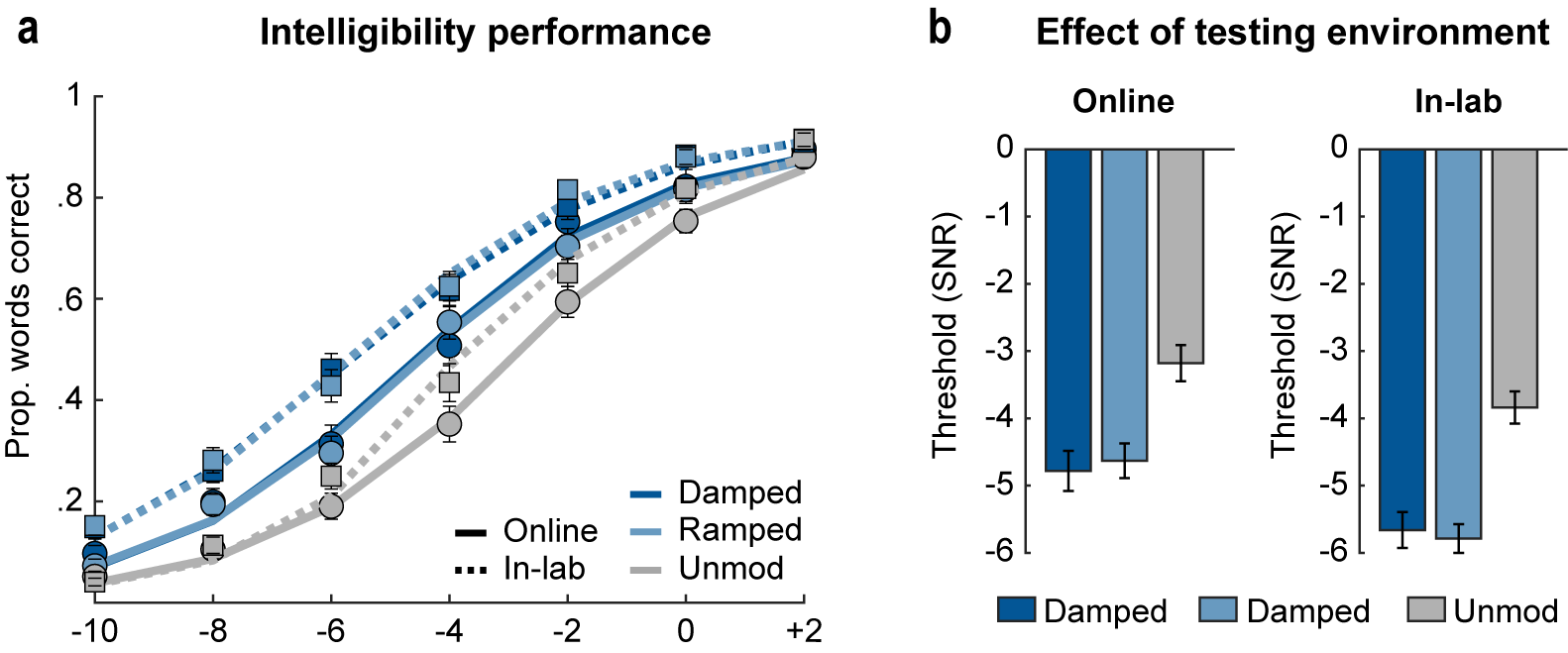


**S1. Effect of testing environment on intelligibility.** **(a)** Mean proportion of correctly reported words plotted as a function of SNR (-10, -8, -6, -4, -2, 0, +2 dB) for online (round markers) and in-lab participants (square markers), and for each envelope condition (damped, ramped, unmodulated). Colored lines (**solid**: online, **dashed**: in-lab) correspond to a logistic function fit to the proportion of correct words reported. **(b)** Mean threshold coefficients from logistic function fits are plotted for the different modulation types (ramped, damped, unmodulated) and testing environments (online, in-lab).

Consistent with Experiment 1, we observed lower thresholds for the ramped and damped maskers compared to the unmodulated masker [effect of modulation type: *F*_2,106_ = 48.53 , *p* = 1.1 × 10^-15^, η^2^_p_ = .48; *t_54_* = -9.06, *p_FDR_* = 6.02 × 10^-12^, r_e_ = .78 ; *t_54_* = -8.86, *p_FDR_* = 6.12 × 10^-12^, r_e_ = .77; Figure S1b], but no difference between ramped and damped thresholds [*p_FDR_* = .964], which suggests better intelligibility when the masker was amplitude modulated than when it was unmodulated. We also observed lower thresholds for participants who completed the intelligibility task in-lab compared to online [effect of testing environment: *F*_1,53_ = 10.03, *p* = .003, η^2^_p_ = .16; Figure S1b]. Age differences could in principle also contribute to these overall threshold differences, because individuals participating in the lab were slightly younger than those participating online, and speech-in-noise thresholds are known to increase with age (see Figure 1 of Experiment 1). Critically, we did not find a significant testing environment × modulation type interaction. This suggests that while in-lab participants performed better on the intelligibility task than online participants (benefit of about -1 dB SNR), the testing environment did not differentially affect performance across the masker conditions, that is, the release from masking effect.

## Assessment of hearing loss in an online sample

In an effort to estimate the level of hearing impairment that may be present in the online samples analyzed in Experiments 1-3, we report the results of a separate online study which assessed hearing impairment via an online implementation of the Digits in Noise test (DIN)^1,2^. DIN thresholds have been shown to strongly correlate with pure-tone average (PTA) thresholds [*r*>.7; 0.5-4 kHz]^3–6^, an objective measure of hearing loss. The DIN test has been adapted and validated for testing in-lab^2,4,6^ and remote testing, such as by telephone^1,7–9^ or a smart phone application^5,10^. Previous work has provided the regression coefficients that describe the linear relation between PTA and DIN thresholds^1^.

Two hundred and sixteen adults (mean: 50 years; age-range: 20-78 years; 108 males 108 females) without self-reported hearing loss, neurological issues, or psychiatric disorders participated in this experiment. Participants were recruited using the same online platform as for Experiments 1-3 (MTurk via CloudResearch Interface). The experiment was conducted online, using custom written JavaScript/html and jsPsych code (Version 6.1.0, a high-level JavaScript library used for precise stimulus control^11^). The experiment code was stored at an online repository (<https://gitlab.pavlovia.org>) and hosted via Pavlovia (<https://pavlovia.org/>).

During the task, participants were asked to listen to digit triplets (e.g., 7-5-9) masked with 12-talker babble noise^12^ and, after the noise and digit triplet ended, type the digits they heard in the order presented. SNR was manipulated by altering the level of the spoken digits relative to the babble noise, and could be one of 26 possible SNRs (range: -15 dB to +15 dB; step size: 1.2 dB). One-hundred digit triplets were pre-generated for each of the 26 SNRs by randomly selecting three different digits ranging from 1 to 9 (onset-to-onset interval: 0.85 s). At the beginning of the experiment, twenty-six digit triplets (one per SNR) were randomly selected for each participant. Each participant completed two practice trials with high SNRs followed by the 26 test trials.

For the data analysis, a trial was only considered correct if all three digits were typed in the order they were presented. A logistic function was fit to the data, and the resulting threshold parameter was used. Older adults had about 1.5 dB higher DIN thresholds compared to younger adults (using the same age ranges as in Experiment 3; Figure S2a).


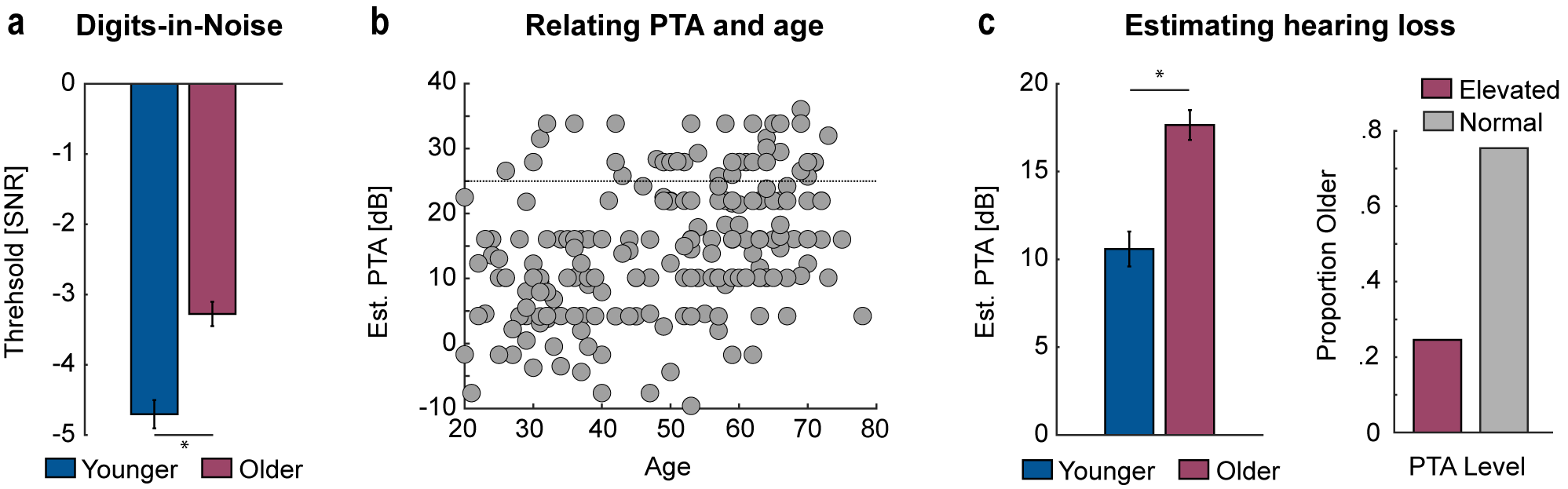


**S2. Estimating hearing loss in online participants. (a)** The relation between age and estimated PTA thresholds (dB) is plotted. The dashed line corresponds to the threshold for elevated PTA thresholds. **(b)** The proportion of adults aged 50 and older with elevated or normal PTA thresholds are plotted. PTA thresholds greater than or equal to 25 dB are considered elevated^13^.

The DIN threshold was subsequently used to derive a PTA (0.5, 1, 2, 4 kHz) estimate for each participant using previously reported regression coefficients (β_1_ = 4.94, β_0_ = 33.84; for slope and intercept, respectively^1^). Figure S2b shows the estimated PTAs as a function of age. PTAs were higher on average in older compared to younger adults (+ 7 dB; using the same age ranges as in Experiment 1; *t*_214_ = 5.41, *p* = 2 × 10^-7^, r_e_ = .35; Figure S2c left panel). This is consistent with mild hearing impairment that has also been reported for in-lab work with community-dwelling adults who are not prescribed with hearing aids (^14–16^).

A more qualitative assessment indicates that the vast majority of participants are predicted to have a PTA in the normal range (<25dB; N = 176; Figure S2b), while approximately 19% of participants are predicted to have an elevated PTA based on their DIN thresholds (N=40). To further investigate the relationship between age and hearing loss in our online sample, we calculated the correlation between age and estimated PTA. We observed that an increase in age was associated with a larger PTA [*r_214_* = .38, *p* = 6.05 × 10^-9^], suggesting that the older adults in the sample were more likely to have hearing issues, as expected. However, of the participants that fit the age criteria for the ‘older’ participant group specified in Experiments 1-3, only 25% are predicted to have elevated thresholds (Figure S2c right panel; 11% for younger adults). If this sample is taken as good representation of the typical distribution of online participants, we would anticipate the vast majority of older and younger online participants without reported hearing issues or hearing aid usage do not have a hearing impairment, while at least a small proportion may have a minor hearing impairment.

# References

1. Smits, C., Kapteyn, T. S. & Houtgast, T. Development and validation of an automatic speech-in-noise screening test by telephone. *Int. J. Audiol.* **43**, 15–28 (2004).

2. Smits, C., Theo Goverts, S. & Festen, J. M. The digits-in-noise test: Assessing auditory speech recognition abilities in noise. *J. Acoust. Soc. Am.* **133**, 1693–1706 (2013).

3. Rudmin, F. Speech reception thresholds for digits. *J. Aud. Res.* **27**, 15–21 (1987).

4. Koole, A. *et al.* Using the digits-in-noise test to estimate age-related hearing loss. *Ear Hear.* **37**, 508–513 (2016).

5. Potgieter, J. M., Swanepoel, D. W. & Smits, C. Evaluating a smartphone digits-in-noise test as part of the audiometric test battery. *South African J. Commun. Disord.* **65**, 1–6 (2018).

6. De Sousa, K. C., Swanepoel, D. W., Moore, D. R., Myburgh, H. C. & Smits, C. Improving Sensitivity of the Digits-In-Noise Test Using Antiphasic Stimuli. *Ear Hear.* **41**, 442–450 (2019).

7. Smits, C. & Houtgast, T. Measurements and calculations on the simple up-down adaptive procedure for speech-in-noise tests. *J. Acoust. Soc. Am.* **120**, 1608–1621 (2006).

8. Smits, C., Kramer, S. E. & Houtgast, T. Speech reception thresholds in noise and self-reported hearing disability in a general adult population. *Ear Hear.* **27**, 538–549 (2006).

9. Smits, C. & Houtgast, T. Results from the Dutch speech-in-noise screening test by telephone. *Ear Hear.* **26**, 89–95 (2005).

10. Brown, L., Mahomed-Asmail, F., De Sousa, K. C. & Swanepoel, D. W. Performance and reliability of a smartphone digits-in-noise test in the sound field. *Am. J. Audiol.* **28**, 736–741 (2019).

11. de Leeuw, J. R. jsPsych: A JavaScript library for creating behavioral experiments in a Web browser. *Behav. Res. Methods* **47**, 1–12 (2015).

12. Bilger, R. C. *Manual for the clinical use of the Revised SPIN test*. (University of Illinois Press, 1984).

13. American National Standards Institute. ANSI S3.6-1996. in *Specification for audiometers.* (New York: American National Standards Institute, 1996).

14. Herrmann, B., Buckland, C. & Johnsrude, I. S. Neural signatures of temporal regularity processing in sounds differ between younger and older adults. *Neurobiol. Aging* **83**, 73–85 (2019).

15. Herrmann, B., Maess, B. & Johnsrude, I. S. Aging affects adaptation to sound-level statistics in human auditory cortex. *J. Neurosci.* **38**, 1989–1999 (2018).

16. Herrmann, B., Maess, B. & Johnsrude, I. S. A neural signature of regularity in sound is reduced in older adults. *Neurobiol. Aging* **109**, 1–10 (2022).
